# Possible Impact of a 12-Month Web- and Smartphone-Based Program to Improve Long-term Physical Activity in Patients Attending Spa Therapy: Randomized Controlled Trial

**DOI:** 10.2196/29640

**Published:** 2022-06-16

**Authors:** Florie Fillol, Ludivine Paris, Sébastien Pascal, Aurélien Mulliez, Christian-François Roques, Sylvie Rousset, Martine Duclos

**Affiliations:** 1 Biomouv SAS Inc Paris France; 2 University of Auvergne Clermont-Ferrand France; 3 Biostatistics Unit (Clinical Research and Innovation Direction) University-Hospital Clermont-Ferrand Clermont-Ferrand France; 4 Physical and Rehabilitation Medicine Paul Sabatier University, Toulouse University Toulouse France; 5 Human Nutrition Unity Centre de Recherche en Nutrition Humaine Auvergne French National Institute for Agriculture, Food and Environment (INRAE) Clermont-Ferrand France; 6 Department of Sport Medicine and Functional Explorations University-Hospital Clermont-Ferrand, G. Montpied Hospital Clermont-Ferrand France; 7 Unité fonctionnelle de Recherche Médecine Clermont University University of Auvergne Clermont-Ferrand France

**Keywords:** physical activity, spa, mobile phone, older adults, internet, exercise, aged, sedentary behavior, quality of life, follow-up studies

## Abstract

**Background:**

Lack of physical activity (PA) and sedentary behaviors are leading risk factors for noncommunicable diseases (NCDs). Web- and smartphone-based interventions are effective in increasing PA in older adults and in patients with NCD. In many countries, spa therapy, commonly prescribed to patients with NCD, represents an ideal context to initiating lifestyle changes.

**Objective:**

This study aimed to evaluate, in patients attending spa therapy, the effectiveness of an intervention combining a face-to-face coaching and, when returning home, a web- and smartphone-based PA program on the achievement of PA guidelines (PAG) 12 months after the end of spa therapy.

**Methods:**

This was a 12-month, prospective, parallel-group randomized controlled trial. Patients were enrolled during spa therapy and randomized 1:1 to intervention or control group who received PA usual advice. From the end of spa therapy, PA, weight, waist circumference, and quality of life of the participants were assessed by phone every 2 months. Primary outcome was meeting PAG (PA ≥600 metabolic equivalent of task) at 12 months. Secondary outcomes were meeting current PAG at 6 months; sedentary time, weight, waist circumference, PA, and quality of life at 6 and 12 months. Objective use data of the web- and smartphone-based PA program were collected. Analytic methods included intention to treat and constrained longitudinal data analyses.

**Results:**

The study sample included 228 participants (n=176, 77.2% females) with a mean age of 62.4 (SD 6.7) years and a mean BMI of 28.2 (SD 4.2) kg/m^2^. Approximately 53.9% (123/228) of the participants were retired. No group differences were found for any baseline variable. At 12 months, the proportion of patients achieving PAG was significantly higher in intervention group than in the control group (81% vs 67% respectively, odds ratio 2.34, 95% CI 1.02-5.38; *P*=.045). No difference between intervention and control group was found neither in achieving PAG at 6 months nor for sedentary time, weight, and waist circumference at 6 and 12 months. Regarding quality of life, the physical component subscale score was significantly higher at 12 months in the intervention group than in the control group (mean difference: 4.1, 95% CI 1.9-6.3; *P*<.001). The mean duration use of the program was 7.1 (SD 4.5) months. Attrition rate during the first 2 months was 20.4% (23/113) whereas 39.8% (45/113) of the participants used the program for at least 10 months.

**Conclusions:**

PA increased in both the intervention group and the control group. However, at 12 months, more participants met PAG in the intervention group compared with the controls. This indicates that the web- and smartphone-based program could have maintained PA in the intervention group. In addition, a spa therapy seems to be an ideal time and framework to implement PA education.

**Trial Registration:**

ClinicalTrials.gov NCT02694796; https://clinicaltrials.gov/ct2/show/NCT02694796

## Introduction

### Background

Lack of physical activity (PA) and excess sedentary behaviors are now recognized as leading risk factors for noncommunicable diseases (NCDs), such as cardiovascular diseases, chronic obstructive pulmonary disease, cancers, and type 2 diabetes, which, taken together, are the primary causes of death worldwide [1]. Insufficient PA, or physical inactivity, is defined as a level of PA below the recommended 150 minutes of moderate PA per week, and sedentary behaviors are defined as “any waking behaviors characterized by an energy expenditure <1.5 metabolic equivalents of task (METs), while in a sitting, reclining or lying posture” [2]. In 2017, of the 41 million NCD-related deaths, 1.26 million were because of insufficient PA [3]. In 2016, >1 in 4 (27.5%) adults worldwide were physically inactive [4]. In 2015, a national survey in France showed that among adults aged 55 to 74 years, 42.2% of women and 28.4% of men did not achieve the recommendation of 150 minutes of moderate PA per week [5]. Engaging people in healthy behaviors such as stopping tobacco use, reducing alcohol consumption, adopting healthy diets, increasing PA, and limiting sedentary time is crucial to tackling the rise of NCDs [1]. Although the health benefits of PA are widely recognized [6], engaging older adults and those with NCDs in long-term lifestyle modifications is very challenging. Although many studies have shown the benefits of PA interventions on the health of patients with NCDs [7], a decrease in PA adherence is frequently observed in the long term, leading to a loss of the acquired health benefits [8].

To maintain adherence to PA, information and communication technologies appear to be promising tools that provide personalized follow-up, real-time feedback, and recommendations. Recent reviews and meta-analyses have found that web- and smartphone-based interventions are effective in increasing PA in the general population [9,10], in older adults [11,12], and in patients with an NCD [13,14]. However, another systematic review [15] suggested that multicomponent interventions, where the use of an app was one of several intervention components such as physical education, provision of PA equipment, parental education, face-to-face counselling, might be more effective than stand-alone app interventions.

In many countries (continental Europe, Japan, China, South America, and North Africa), a course of spa therapy is accepted as treatment by the health insurance system and is commonly prescribed to patients with chronic diseases such as rheumatic conditions, respiratory diseases, and skin diseases and patients convalescing from cancer, as well as to those who are overweight or obese. In France, the 3-week courses of therapy delivered in spa centers are reimbursed by the national social security. The context and environment of a stay in a spa therapy center have been shown to be conducive to educating patients about their disease and initiating lifestyle changes, including increasing PA, through patient therapeutic education in PA programs [16-20].

### Objectives

We hypothesized that an intervention combining individual face-to-face coaching during spa therapy with a subsequent 12-month web- and smartphone-based PA program would improve PA in patients undergoing spa therapy. The main objective of this study was to evaluate the effectiveness of the intervention compared with the usual advice (ie, standard advice on PA provided during spa therapy) on the achievement of PA recommendations 12 months after the end of spa therapy. Secondary outcomes were to evaluate, throughout the 12-month follow-up, at 6 and 12 months, the effectiveness of the intervention on PA, sedentary time, weight, waist circumference, quality of life of the patients, and engagement with the program (the number of performed PA sessions and frequency of use of the program).

## Methods

### Study Design

This was a 12-month, prospective, parallel-group, open, multicenter, single-blinded randomized controlled trial (RCT) that enrolled patients attending a 3-week spa therapy treatment. It evaluated the effectiveness of individual face-to-face PA coaching during the stay at the spa therapy facility followed by a 12-month web- and smartphone-based PA program, including a connected wrist pedometer and a connected weighing scale. Participants were randomized 1:1 to either the intervention group or the control group. The participants were enrolled in 1 of 8 French spa therapy facilities: Amélie-les-Bains, Bourbon-Lancy, Brides-les-Bains, Le-Boulou, Chaudes-Aigues, Eugénie-les-Bains, Vals-les-Bains, and Vichy.

### Participants and Recruitment

Enrollment and follow-ups were conducted between September 2015 and December 2017. Patients were recruited through posters and flyers displayed in spa therapy facilities and spa physicians’ surgeries. A PA instructor was allocated to each spa center to prescreen all potential patients and evaluate their eligibility. Spa physicians participating in the study could also refer their patients to the PA instructor for prescreening. The inclusion criteria were as follows: age of 50 to 79 years, diagnosis of a stabilized chronic disease (cardiovascular disease, obesity, type 2 diabetes, chronic obstructive pulmonary disease, rheumatic conditions, and breast cancer), BMI between >19 kg/m^2^ and <35 kg/m^2^, undertaking PA for <150 minutes per week, and having smartphone access to the internet. Exclusion criteria included having a cardiac pacemaker, nonstabilized chronic disease, locomotor disability, evolving metastatic cancer, or a contraindication to PA. Eligible participants underwent a medical examination with the spa physician, who after checking that they could safely follow the study protocol, included them in the trial after the participants provided informed consent. Randomization of the participants to the intervention or control group was stratified by gender and center (thermal spa resort) and performed by the spa PA instructor using a centralized secured management system, REDCap (Research Electronic Data Capture; Vanderbilt University).

### Intervention and Control

The intervention comprised a 1-hour individual coaching session with a PA instructor during the 3-week spa therapy stay in 1 of the 8 spa care facilities and then access to the web- and mobile-based PA program and associated connected devices for the 12 months following the end of the spa therapy. All PA instructors received the same training and used the same material. The first part of the consultation aimed to introduce or remind the participants of the benefits of PA for health and disease management. The PA instructor provided advice on how to reach the recommended level of PA and examples of PA adapted to the patient’s particular condition. Subsequently, the PA instructor presented the automated web and mobile-based PA program (Thermactive, BIOMOUV SAS Inc) together with the use of connected devices (weighing scales and wrist pedometer; Multimedia Appendices 1 and 2). The PA instructor downloaded the mobile app onto the patient’s smartphone and showed him or her how to log into the mobile app and connect and use the weighing scale. The PA instructor also explained access to the website and showed participants the main functionalities of the program. The patients were registered in the program by the PA instructor who completed a web-based questionnaire to determine the patient’s PA profile: age, weight, height, physical fitness (endurance, strength, flexibility, and balance measured by the PA instructor), PA, joint disabilities, and pathology. The patient also declared his availability for PA sessions, his PA preferences, and his sports material (such as dumbbells, yoga mats, bands, and wrist weights). Participants in the intervention group followed the web- and mobile-based PA program for 12 months from the end of their 3-week stay in the spa therapy center.

The automated program aimed to help patients achieve recommended levels of PA in 2 ways: by proposing personalized and structured PA sessions and by increasing daily PA (number of steps). The PA sessions were automatically generated based on the patient’s profile. To generate personalized PA sessions, an algorithm was developed to select and associate exercises from a database of >1500 different exercises. Each exercise was classified according to its nature (aerobic, strengthening, and balance), part of the body concerned (leg, arm, and trunk), exercise intensity, and duration. The algorithm selected exercises appropriate to a patient’s physical capacity and availability and constructed a PA session adapted to the patient. Each PA session comprised 3 phases: a 5-minute warm-up period; either 10 to 35 minutes of exercise to develop muscle strength and flexibility or 10 to 50 minutes of endurance during walking or cycling (mixing continuous and intermittent effort); and finally, a 5-minute recovery phase comprising stretching and relaxation or a return to calm after walking sessions. The PA sessions were either automatically compiled videos or PDF files. The program of PA sessions followed international guidelines regarding the number of sessions per week, resting periods, type of exercise (resistance and endurance), duration, and intensity of each exercise [21]. For each participant, their PA sessions evolved during the course of the intervention taking into account the number of PA sessions completed (recorded by the patient) and any difficulty perceived at the end of the PA sessions (collected using a Borg scale [22]). To increase daily PA, the program generated a daily goal of the number of steps to be achieved based on data from the pedometer over 7 consecutive days. The achievement of these goals determined the subsequent goals, and every day, participants received a notification on their mobile app about the achievement of their personal goals. They also received emails about new PA sessions available on the website and emails reminding them whether a PA session had not been performed and inviting them to do it when possible. Participants had the possibility to record or add activities on the mobile app, which were not planned in the program, such as walking, cycling, swimming, or fitness sessions. The website and the mobile app also allowed participants to record their daily PA and amount of sedentary time to visualize their evolution over time.

Patients allocated to the control group received the usual advice on PA and a booklet providing advice and examples of PA suited to their pathology. At the end of the 12-month follow-up period, the patients included in the control group received free connected devices and access to the Thermactive program for 12 months.

### Measurements and Follow-up

Data collected during the study and follow-up were recorded using an electronic case report form in a centralized secured management system, REDCap.

Demographic variables of the participants, including sex, age, weight, waist circumference, highest level of formal education (high school or less and higher education), occupation (nonworking [retired or unemployed], manager [artisan or intellectual profession], and employee [employee, intermediate occupation, and worker]), condition treated by spa therapy, medical-surgical and family history, medical treatments, physical fitness, PA, and quality of life were collected at baseline (month 0 [M0]) by the PA instructor. PA was assessed using the validated International Physical Activity Questionnaire (IPAQ)-short version [23]. The IPAQ measures the frequency (days per week) and duration (minutes) of PA during the past 7 days in the following domains: work, transportation, work at home, and leisure activities [23]. Different levels of PA (walking, moderate, vigorous, and total) were calculated and expressed in METs minutes per week (a product of PA intensity and duration). PA was classified as low (<600 MET minutes per week), moderate (600-3000 METs), or high (>3000 METs) [23]. Meeting current PA guidelines (PAG) was defined as a total PA of ≥600 METs [23].

At inclusion (M0), physical fitness was evaluated in both groups using validated physical fitness field tests from *Eurofit for Adults* [24] and the *Senior Fitness Tests* [25], with the 6-minute walk test to assess cardiorespiratory fitness (endurance), the arm curl test, the 30-second chair stand test for muscle strength, the lateral side–bending test for flexibility and patients’ balance by the one-leg standing test, and the timed up and go test for balance. Quality of life was assessed using the Short Form Health Survey-12 (SF-12; version 2) [26]. The SF-12 assesses limitations in role functioning with 12 items. It consists of 2 subscales measuring physical health (physical component subscale [PCS]) and mental health (mental component subscale). The presence and severity of different impairments over the past 4 weeks are rated. Subscale scores can vary between 0 and 100, with higher scores indicating less impairment or greater health well-being.

From the end of the 3-week spa therapy, PA, body weight, waist circumference, and quality of life of the participants in both groups were assessed at month 2 (M2), month 4, month 6 (M6), month 8, month 10, and month 12 (M12) by interviewers (masked to the participant’s randomization group) by phone. Data were collected every 2 months to avoid a loss to follow-up and to allow more precise measurement of change in outcome over time.

To limit missing data, participants were contacted 3 times for each follow-up phone interview. First, the participants were contacted by email to plan the phone interview; in case of no answer, an SMS text message was sent to his or her cell phone within 7 days, and after failing to respond within 3 days of the SMS text message, he or she was contacted directly by phone. The interviewer tried to contact nonresponders for 1 month after the theoretical follow-up date.

### Outcomes

The primary outcome was meeting the current PAG at 12 months after the end of spa therapy, defined as reporting total PA ≥600 METs [23] measured by the IPAQ short form.

Secondary outcomes were meeting the current PAG at 6 months after the end of spa therapy; sedentary time, weight, waist circumference, PA, and quality of life at 6 and 12 months; and changes in these indicators evaluated every 2 months during the 12-month follow-up.

The use of the program was evaluated by the number of connections to the Thermactive website, number of PA sessions conducted (structured PA sessions+recorded PA sessions), and number of months for which use of the program was maintained.

### Sample Size

With a risk of 0.05, a power (1-b) of 0.90, and assuming a detectable difference in patients meeting the PAG of 15% between the 2 groups [27], the sample size required was 462, with 231 participants in each study arm.

### Statistics

Continuous variables were described as mean (SD or 95% CI) or median (IQR). The normal distribution of continuous variables was checked using the Shapiro-Wilk test. To compare between-group differences, a Student *t* test (2-tailed) was used for variables with normal distribution; otherwise, the Mann-Whitney test was used. Categorical variables are presented as frequencies and percentages and were compared between groups using a chi-square test. To test effectiveness, the data were analyzed using intention-to-treat principles [28]. As all randomized patients were included in the analyses and considering that assessment every 2 months should limit the loss to follow-up, attrition was not considered to increase the sample size [29]. To compare between-group differences from baseline for repeated outcomes, a constrained longitudinal data analysis (CLDA) was used. This mixed model is a constrained full-likelihood approach, whereby both the baseline and postbaseline values are modeled as dependent variables (the constrained model assumes that both the baseline and postbaseline measurements are jointly multivariate and normally distributed as the baseline value is treated as part of the response vector), and the true baseline values are constrained to be the same for the 2 treatment groups. Such methods based on maximum likelihood are consistent under the *missing at random* assumption. This model allows the inclusion of patients for whom either the baseline or postbaseline measurements are missing, thereby increasing efficiency [30]. Hence, this analysis provides an adjustment for the observed baseline difference in estimating the intervention effects. Time was treated as a categorical variable so that no restriction was imposed on the trajectory of the means over time. In addition to adjusting for baseline covariates, the analysis model was also adjusted for the intervention, time, sex, and interaction of time and intervention. Random effects at the patient and center levels were also included. The results are expressed as odds ratios (ORs) with 95% CI and *P* values for categorical variables and as differences in mean change from baseline to 1 year with 95% CI for continuous variables. All statistical tests were 2-sided, and *P*<.05 was considered statistically significant. Data were analyzed using Stata 12.

### Safety

All serious adverse events (AEs) were recorded and notified to the French clinical trials pharmacovigilance system.

### Ethics Approval

The trial, funded by Association Française pour la Recherche Thermale (grant number 2015-02), a nonprofit independent organization, was approved by the National Agency for the Safety of Medicine and Health Products and the regional ethics committee (Comité de Protection des Personnes Sud-Est N 6; registration number: CPP AU1196; registration number IDRCB:2015-A00855-44) and registered at ClinicalTrials.gov (NCT02694796) before enrollment of the participants began.

## Results

### Patients

**Figure 1:**
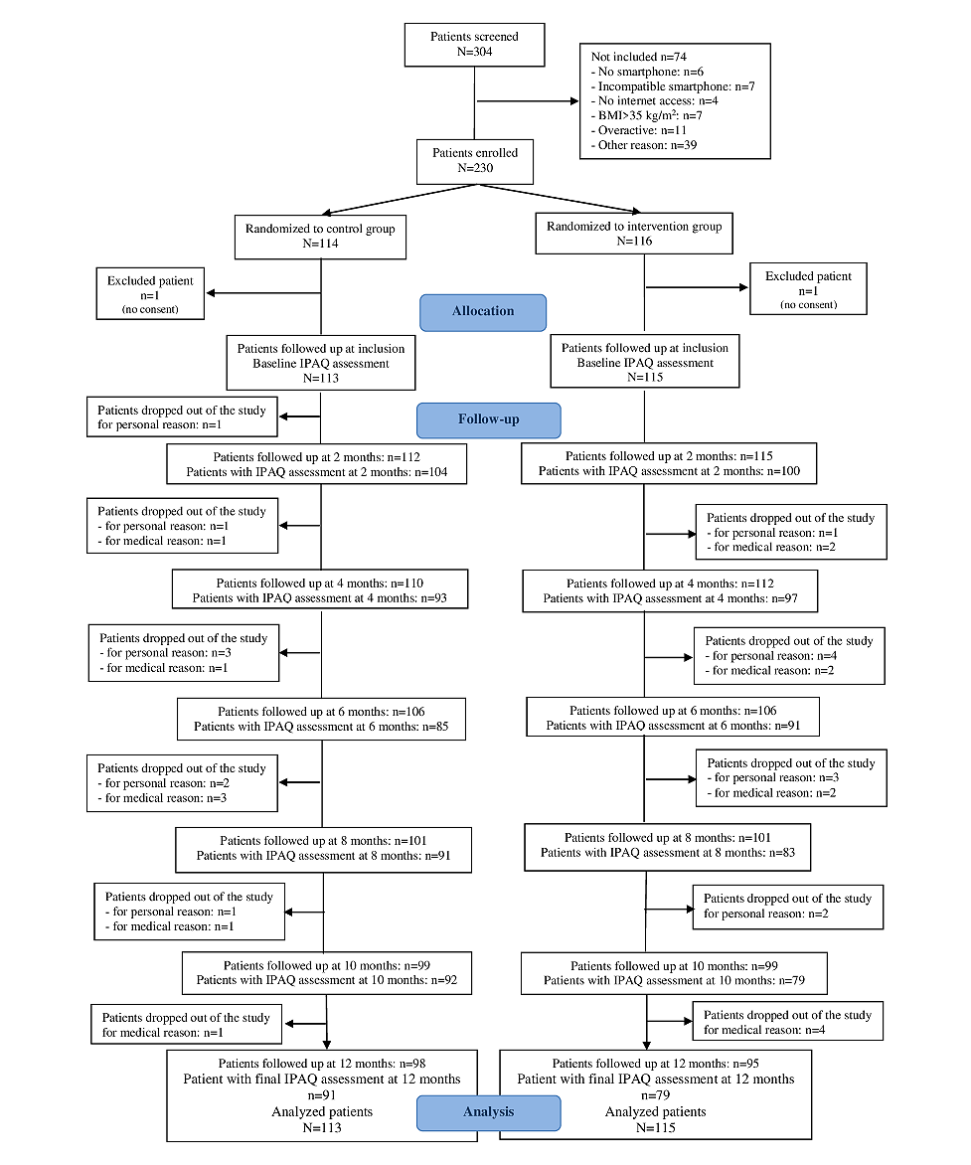
CONSORT (Consolidated Standards of Reporting Trials) flowchart. IPAQ: International Physical Activity Questionnaire.

Recruitment was conducted from September 2015 to December 2016. Of the 304 patients screened, 230 (75.6%) were enrolled and randomly assigned to either the control group (n=114, 49.6%) or intervention group (n=116, 50.4%; Figure 1). After randomization, 0.9% (2/230) of patients (1 in each group) withdrew their participation; thus, a total of 228 patients were included in the analyses. Patient characteristics are presented in Table 1. More than 3 participants out of 4 were women (176/228, 77.2%). The mean age of the sample was 62.4 (SD 6.7) years, and 53.9% (123/228) of the participants were retired. The 2 groups did not differ in any variable recorded at baseline.

**Table 1:** Baseline characteristics of the participants in the control and intervention group (N=228).

Characteristics		Control group (n=113)	Intervention group (n=115)	Total
Female, n (%)		86 (76.1)	90 (78.3)	176 (77.2)
Age (years), mean (SD)		62.3 (6.9)	62.6 (6.6)	62.5 (6.7)
Weight (kg), mean (SD)		76.3 (15.1)	77.0 (14.3)	76.7 (14.6)
BMI, mean (SD)		28.3 (4.4)	28.2 (4.0)	28.3 (4.2)
Waist circumference (cm), mean (SD)		96.7 (13.6)	95.0 (12.4)	95.8 (13.0)
**Educational level, n (%)**				
	High school or less	62 (54.9)	55 (47.8)	117 (51.3)
	Higher education	51 (45.1)	60 (52.2)	111 (48.7)
**Occupation, n (%)**				
	Nonworking (retired, unemployed, housewife or househusband, disability, or long-term leave)	77 (68.1)	74 (64.3)	151 (66.2)
	Manager (artisan, trader, senior executive, or intellectual profession)	18 (15.9)	16 (13.9)	34 (14.9)
	Employee (intermediate occupation or worker)	17 (15)	24 (20.9)	41 (18)
**Indication for** **spa** **treatment, n (%)**				
	Arthrosis	82 (72.6)	83 (72.2)	165 (72.4)
	Cardiovascular diseases	14 (12.4)	11 (9.6)	25 (11)
	Obesity	17 (15)	19 (16.5)	36 (15.8)
	Diabetes (type 1 and type 2)	8 (7.1)	12 (10.4)	20 (8.8)
	COPD^a^	2 (1.8)	1 (0.9)	3 (1.3)
	Cancer	3 (2.7)	4 (3.5)	7 (3.1)
	Other	26 (23)	22 (19.1)	48 (21.1)
**Physical fitness, mean (SD)**				
	Resting heart rate (beats per minute)	70.1 (9.8)	71.1 (10.7)	70.6 (10.3)
	6-minute walk test (minutes)	463 (97.6)	464.6 (94.6)	463.8 (95.9)
	Arm curl test (number of flexions)	22 (6.9)	22.1 (7.2)	22.0 (7.0)
	30-second chair stand test (number of up-and-down)	14.3 (4.1)	13.8 (4.4)	14.0 (4.2)
	Lateral side–bending test (right side; cm)	15.9 (4.2)	15.2 (3.5)	15.6 (3.9)
	Lateral side–bending test (left side; cm)	16 (4.3)	15.4 (3.7)	15.7 (4.0)
	One-leg standing test (seconds)	6.2 (9.0)	6.0 (6.3)	6.1 (7.8)
	Timed up and go test (seconds)	6.2 (1.5)	6.2 (1.7)	6.2 (1.6)
**PA^b^ (IPAQ^c^; MET^d^ minutes per week), median (IQR)**				
	Continuous score for vigorous intensity	0 (0-960)	0 (0-480)	0 (0-960)
	Continuous score for moderate intensity	120 (0-240)	240 (0-360)	130 (0-360)
	Continuous score for walking	198 (66-396)	198 (66-346.5)	198 (66-396)
	Continuous score for overall activity	396 (198-664)	419 (238-720)	396 (198-686)
**Sedentary time (IPAQ**; **minutes), median (IQR)**				
	Time spent sitting on a week day	300 (240-420)	360 (270-480)	360 (240-480)
	Time spent sitting on a weekend day	300 (240-360)	300 (240-360)	300 (240-360)
	Time spent watching television on a week day	120 (120-180)	120 (120-180)	120 (120-180)
	Time spent watching television on a weekend day	120 (120-240)	120 (120-180)	120 (120-180)
	Time spent in front of computer or tablet on a week day	120 (60-180)	120 (60-240)	120 (60-210)
	Time spent in front of computer or tablet on a weekend day	60 (30-120)	60 (45-150)	60 (30-120)
**Quality of life (SF-12^e^; 0-100), mean (SD)**				
	Physical health (PCS^f^)	43.2 (8.5)	43.3 (8.5)	43.2 (8.5)
	Mental health (MCS^g^)	47.0 (9.5)	48.1 (8.9)	47.6 (9.2)

^a^COPD: chronic obstructive pulmonary disease.

^b^PA: physical activity.

^c^IPAQ: International Physical Activity Questionnaire.

^d^MET: metabolic equivalent of task.

^e^SF-12: Short Form Health Survey-12.

^f^PCS: physical component subscale.

^g^MCS: mental component subscale.

### Primary Outcome

**Figure 2:**
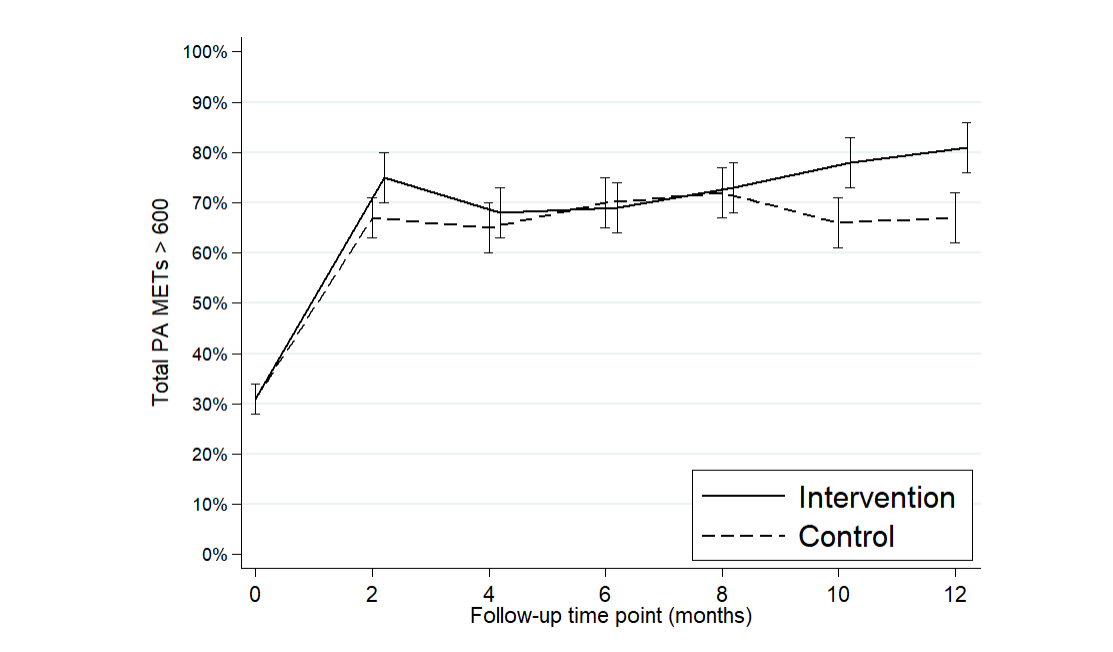
Change in the percentage of PA guidelines achievement (total PA MET≥600) marginal values according to constrained longitudinal data analysis model for each group over time. MET: metabolic equivalent of task; PA: physical activity.

The change in the percentage achieving PAG marginal values according to CLDA modeling for each group is presented in Figure 2, and the statistical comparison between the groups for PAG achievement is shown in Table 2. The achievement of PAG significantly increased in both groups from M0 to M12 (Table 2), with the greatest increase occurring between M0 and M2 (Figure 2). At 12 months, the proportion of patients achieving PAG was significantly higher in the intervention group than in the control group (64/79, 81% vs 61/91, 67%, respectively; Figure 2; OR 2.34, 95% CI 1.02-5.38; *P*=.045; Table 2). The CLDA analysis also showed that significantly fewer women achieved PAG than men (*P*=.005; Table 2).

**Table 2:** Constrained longitudinal data analysis model of the achievement of physical activity guidelines (total physical activity metabolic equivalents of task ≥600) over time.

Characteristics	Odds ratio (95% CI)	*P* value
Female	0.52 (0.33-0.83)	.005
Inclusion visit (month 0)	N/A^a^	N/A
2-month visit (month 2)	6.3 (3.55-11.17)	<.001
4-month visit (month 4)	5.49 (3.05-9.87)	<.001
6-month visit (month 6)	7.41 (3.97-13.85)	<.001
8-month visit (month 8)	8.37 (4.5-15.55)	<.001
10-month visit (month 10)	5.79 (3.2-10.48)	<.001
12-month visit (month 12)	6.29 (3.45-11.46)	<.001
Intervention group×month 2	1.58 (0.77-3.23)	.21
Intervention group×month 4	1.18 (0.58-2.41)	.65
Intervention group×month 6	0.95 (0.45-2.01)	.89
Intervention group×month 8	1.04 (0.47-2.26)	.93
Intervention group×month 10	2.12 (0.95-4.74)	.07
Intervention group×month 12	2.34 (1.02-5.38)	.045

^a^N/A: not applicable.

### Secondary Outcomes

#### PA and Sedentary Times

**Figure 3:**
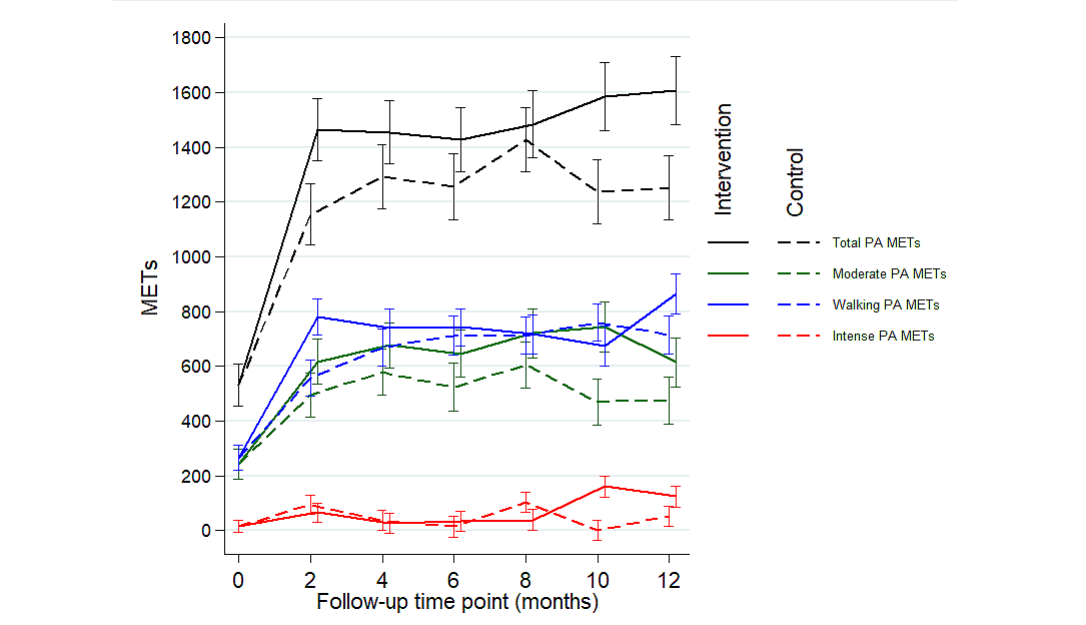
International Physical Activity Questionnaire scores for total, moderate, intense, and walking physical activity margin values according to constrained longitudinal data analysis model for each group over time. MET: metabolic equivalent of task; PA: physical activity.

At 6 months follow-up, the achievement of PAG did not differ between the intervention and control groups (63/91, 69.2% and 59/84, 70.2% of patients reached the PAG, respectively; Figure 2; OR 0.95, 95% CI 0.45-2.01; *P*=.89; Table 2). Regarding the PA level (Figure 3), the IPAQ score of total PA at M12 was significantly higher in the intervention group than in the control group (intervention group total PA 1618 METs, 95% CI 1491-1744 METs vs control group total PA 1275 METs, 95% CI 1140-1385 METs; *P*=.04), whereas no significant difference was observed at M6 (intervention group total PA 1427 METs, 95% CI 1303-1564 METs vs control group total PA 1274 METs, 95% CI 1146-1392 METs; *P*=.30).

**Figure 4:**
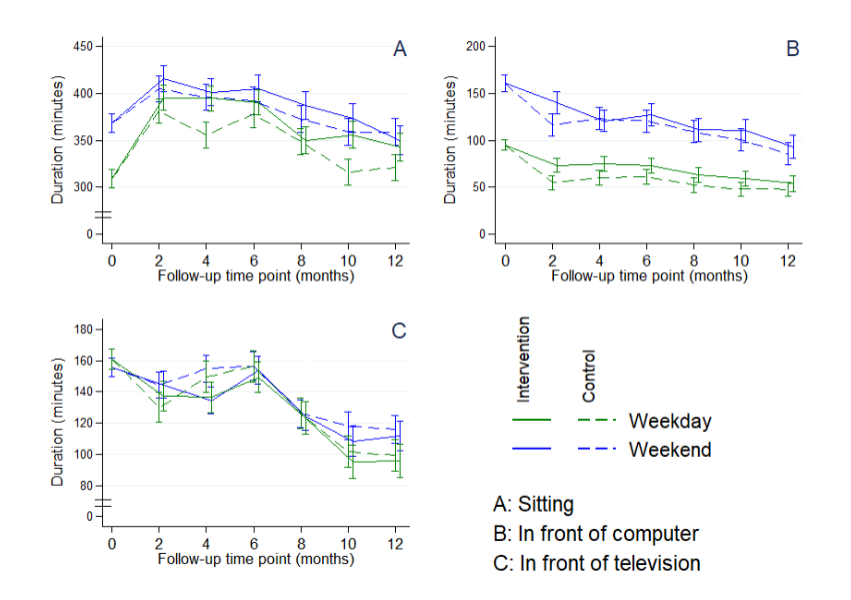
Sedentary times marginal values according to constrained longitudinal data analysis model for each group over time.

There were no statistically significant differences between the 2 groups at M6 or M12 regarding the IPAQ scores for walking, moderate, and vigorous PA (Figure 3) or for sitting time or time spent in front of a screen (television or computer) during weekdays or weekends (Figure 4).

Nevertheless, the time spent in front of a screen (computer or television) decreased significantly over the follow-up in both the groups during both weekdays and weekends (Table 3).

**Table 3:** Change in time spent in front of screens (computer or television) for control and intervention groups pooled.

Duration of time	Change between month 12 and month 0, mean (SE; 95% CI)	*P* value
Time spent in front of a computer during the week (minutes)	−75.2 (10.2; −95.3 to −55.2)	<.001
Time spent in front of a computer during the weekend (minutes)	−47.2 (7.8; −62.6 to −31.8)	<.001
Time spent in front of a television during the week (minutes)	−39.7 (9.3; −58.0 to −21.4)	<.001
Time spent in front of a television during the weekend (minutes)	−61.9 (10.8; −83.0 to −40.7)	<.001

#### Body Weight and Waist Circumference

**Figure 5:**
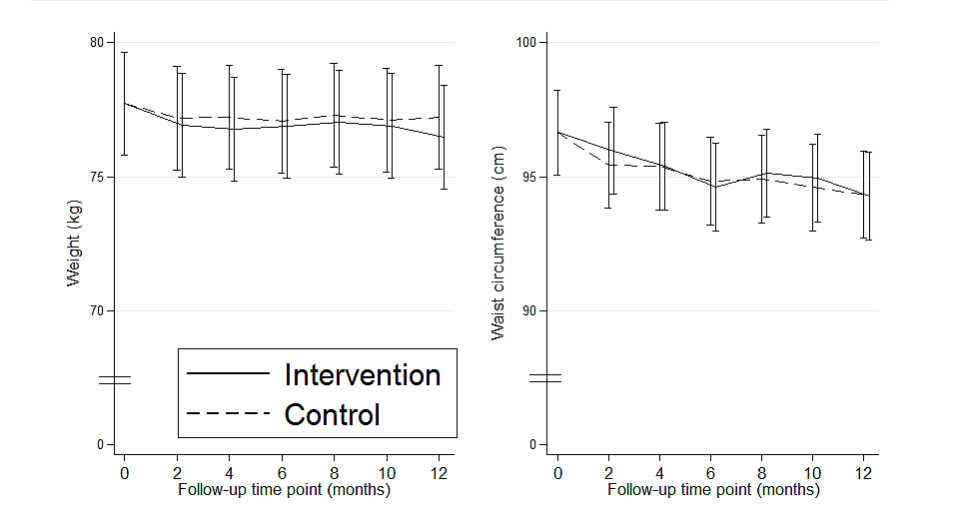
Weight and waist circumference marginal values according to constrained longitudinal data analysis model for each group over time.

There was no statistically significant difference between the 2 groups for body weight or waist circumference at M6 and M12 (Figure 5). However, the mean waist circumference for the 2 groups had significantly decreased at 6 months by 1.9 cm (95% CI −3.0 to −0.8 cm; *P*=.001) and at 12 months by 2.4 cm (95% CI −3.5 to −1.3 cm; *P*<.001).

#### Quality of Life

**Figure 6:**
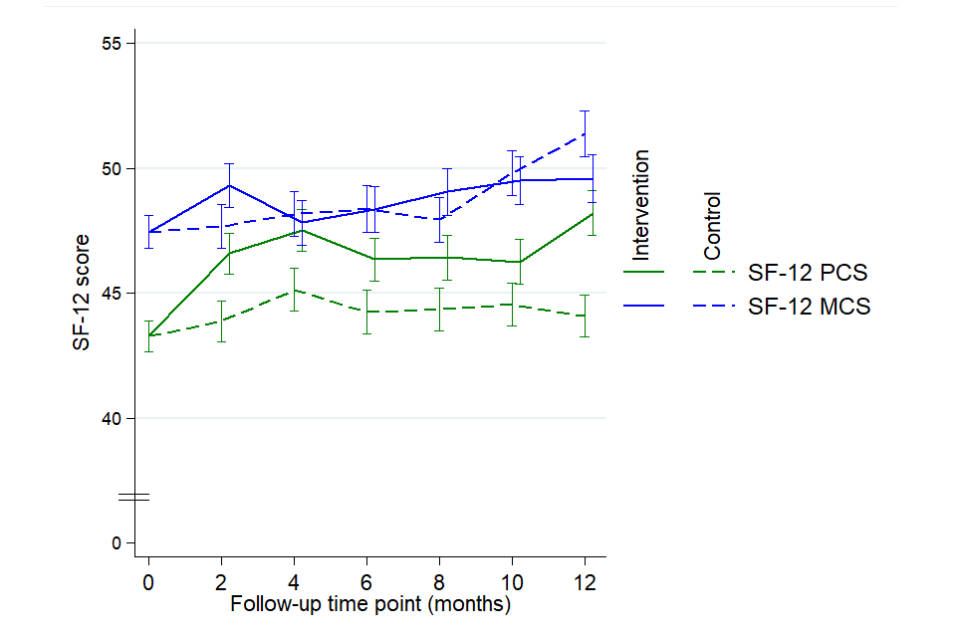
SF-12 scores (PCS and MCS) marginal values to constrained longitudinal data analysis model for each group over time. MCS: mental component subscale; PCS: physical component subscale; SF-12: Short Form Health Survey-12.

The quality of life assessment showed that the PCS score was significantly higher at M12 in the intervention group than in the control group (Figure 6; mean difference at M12 4.1, 95% CI 1.9-6.3; *P*<.001). At M6, the PCS score tended to be higher in the intervention group than in the control group (PCS score 2.1, 95% CI 0.0-4.3; *P*=.06). There were no statistically significant differences between the 2 groups in the mental component subscale score at M6 or M12 (Figure 6).

#### Use of the Program

Monitoring of the program use results is presented in Table 4. The patients used the program for an average of 7.1 (SD 4.5) months. Approximately 20.4% (23/113) dropped out of the program before 2 months of use; however, 39.8% (45/113) of the participants used the program for ≥10 months (Table 4). Among the participants, 62.8% (71/113) had at least one structured PA session.

**Table 4:** Use of the program (N=115).

Characteristics		Intervention group
**Logging into the program**		
	Patients who logged into the program at least once, n (%)	113 (98.3)
	Total number of log-ins into the program, N	16,325
	Number of log-ins by patients, mean (SD)	143.2 (179.4)
	Number of log-ins by patients, median (IQR)	76 (24.3-208.8)
**Duration of program use**		
	Duration of use (months), mean (SD)	7.1 (4.5)
	Patients who used the program for <2 months, n (%)	23 (20.4)
	Patients who used the program for 2 to 4 months, n (%)	14 (12.4)
	Patients who used the program between 4 and 6 months, n (%)	13 (11.5)
	Patients who used the program between 6 and 8 months, n (%)	8 (7.1)
	Patients who used the program between 8 and 10 months, n (%)	10 (8.8)
	Patients who used the program for >10 months, n (%)	45 (39.8)
**Total PA^a^ sessions (recorded+structured)**		
	Patients who conducted at least one PA session, n (%)	81 (71.7)
	Total number of PA sessions conducted, N	2588
	Number of PA sessions conducted, median (IQR)	16 (3-47)
**Structured PA sessions**		
	Patients who conducted at least one structured PA session, n (%)	71 (62.8)
	Total number of structured PA sessions conducted, N	1836
	Number of structured PA sessions conducted, median (IQR)	8 (2-34)
	Patients who conducted <1 structured PA session by month of use, n (%)	25 (35.2)
	Patients who conducted 1 to 4 structured PA sessions by month of use, n (%)	26 (36.6)
	Patients who conducted 4 to 8 structured PA sessions by month of use, n (%)	16 (22.5)
	Patients who conducted >8 structured PA sessions by month of use, n (%)	4 (5.6)

^a^PA: physical activity.

#### Safety

AEs recorded during the study are presented in Table 5. None of the severe AEs were attributed to the intervention. One patient reported an aggravation of lymphedema in the left arm because of wearing a wrist pedometer. This adverse effect was resolved by physiotherapy.

**Table 5:** Adverse events recorded during the follow-up (N=228).

Adverse events	Control group (n=113), n (%)	Intervention group (n=115), n (%)
Adverse events	102 (49.6)	70 (60.9)
Severe adverse events	13 (11.5)	11 (9.6)
Increased arthrosis	2 (1.8)	0 (0)
Hospitalizations or care for a disorder unrelated to the spa indication	11 (9.7)	11 (9.6)

## Discussion

### Principal Findings and Comparison With Prior Work

This RCT aimed to assess the effectiveness of an intervention, including an initial face-to-face coaching and a web- and mobile-based PA program, to meet PAG among patients attending a 3-week spa therapy treatment. The results showed that significantly more participants met the PAG at the 12-month follow-up in the intervention group than in the controls; however, no difference was observed between the 2 groups for reaching PAG at 6 months. The intervention significantly improved the physical component of the quality of life at 12 months. Sedentary times and waist circumference were significantly reduced in both groups at 6 and 12 months of follow-up without significant differences between the groups.

The level of PA increased in both groups but was significantly higher at 12 months in the intervention group. The increase in PA in the control group might be explained by the usual advice on PA and lifestyle changes provided during the 3-week spa therapy by health care professionals. Indeed, a number of studies have shown that the context and environment of spa treatments represent an opportunity to educate patients on their chronic diseases and initiate behavioral changes [16-20], such as PA.

Our analyses showed that the effect of usual advice on PA in the control group was the highest during the first 2 months after the spa therapy; subsequently, this tended to stabilize and finally slightly decreased after 8 months. Although the PA in the intervention group followed the same dynamic for the first 8 months, it increased after 8 months and became significantly higher at 12 months.

The maintenance of the level of PA to reach the PAG at 12 months in the intervention group could be explained by the web- and mobile-based PA program. This result is in line with the results observed in other RCTs aimed at improving PA among older adults using web-based PA interventions [31,32]. A systematic review and meta-analysis evaluated the effects of eHealth interventions on promoting PA in older adults [12]. The results of this meta-analysis showed that the effects of the eHealth intervention (vs controls) on PA time measured by questionnaires and objective wearable devices on energy expenditure and step counts were all significant with minimal heterogeneity.

Our findings also highlight that the intervention significantly improved the physical component of quality of life at 12 months, which is consistent with the increase in physical abilities because of the improvement in PA level. Limited studies have reported on the effect of web- or mobile-based PA interventions on quality of life among older adults. A randomized control trial that included 235 participants indicated that after 3 months, an internet-based intervention aimed at increasing PA significantly improved the quality of life of inactive older adults [33]. Another study conducted by Irvine et al [34] also showed a significant improvement in the SF-12 PCS among sedentary older adults aged >55 years who engaged in a web-based PA program.

Our results indicate that waist circumference was significantly reduced in both groups at 6 and 12 months of follow-up without a significant difference between the groups.

A meta-analysis [35], including 31 RCTs, emphasized that internet-based interventions showed a significant reduction in waist circumference (mean change −2.99 cm, 95% CI −3.68 to −2.30 cm; *I*^2^=93.3%) compared with minimal interventions such as information-only groups. Our findings indicate a similar mean change in waist circumference in the 2 groups (−2.4 cm; 95% CI −3.5 to −1.3 cm). Therefore, this reduction did not seem to be explained by the intervention. The inclusion in a research study and the focus on their medical conditions should motivate them to adopt better health behaviors. The time spent sitting was higher at M2, month 4, and M6 in both groups than that at baseline. This could be because of fatigue related to the increase in PA [36], which induced compensatory time spent being sedentary, probably at the expense of light PA (unassessed by the IPAQ questionnaire, but which can represent most PA in older adults). This hypothesis should be confirmed in future studies.

Our results also indicate that men were more likely to successfully reach the PAG than women. The present findings are consistent with those of previous studies. Blanchard et al [37] evaluated PA levels in patients with heart disease over 12 months (with or without cardiac rehabilitation) and showed a more pronounced decline in PA over time in women than in men. Jenkins and Gortner [38] specifically examined gender disparity in PA in people living with heart disease who did not receive cardiac rehabilitation. The results showed that men walked significantly more than women at 1, 2, 6, and 12 months after hospitalization. However, analyzing the determinants of parameters that establish which factors predict which participants are successful in reaching PAG was not a part of our research question. Such a determinant analysis will be performed in forthcoming studies and will address different research questions with the ultimate aim of better targeting different populations.

### Limitations and Strengths

The effect of the intervention on maintaining long-term PA and reaching PAG needs to be viewed cautiously as, despite an extension of the enrollment period, the a priori sample size was not met. Two main reasons explain the difficulties in including participants in the trial. First, it appeared that many patients with a web connection and smartphone were already meeting the PAG. Second, we encountered difficulties in the recruitment of qualified PA instructors who played an essential role in the prescreening of participants and face-to-face coaching of the intervention group.

Another limitation of our trial is the self-reported assessment measures, making them potentially subject to social desirability bias [39]. Furthermore, the Hawthorne effect [40] (referring to a tendency in some individuals to alter their behavior in response to their awareness of being observed) along with contamination bias could also affect the magnitude of the differences observed in the results. However, the contamination bias cannot call into question our main result as it reduced the size of the difference between the 2 groups. Therefore, we can hypothesize that without contamination bias, the difference between the 2 groups would have been greater.

The Hawthorne effect and the repeated assessment of outcomes every 2 months could motivate participants to become more active, leading them to overestimate the report of PA and consequently bias our findings. Although this bias could have occurred in both the control and intervention groups and, therefore, would not bias the comparison between the 2 groups, the proportion of participants achieving PAG might be overestimated. Moreover, we cannot exclude that participants in the intervention group may be influenced by the expectation that they will perform better as they received the promising PA program, especially at the end of the program, resulting in an overestimation of their PA level.

A greater number of patients was assessed at M12 in the control group (91/113, 80.5%) than in the intervention group (79/115, 68.7%). One of the reasons for this higher compliance of the control group may be the promise to have free access to the program at the end of the follow-up.

The use of the program can be considered satisfactory as patients used the program for an average of 7.1 (SD 4.5) months; 78.3% (90/115) of the patients used the program for at least 2 months and 39.1% (45/115) for at least 10 months. Approximately 61.7% (71/115) of patients reported engaging in structured PA sessions (median 8 sessions), emphasizing the clear interest of participants in the value of the program, as well as its acceptability and usability. Indeed, the attrition rate for web and smartphone interventions in PA is often quite high [41] (ranging from 30% [42] to 80% [43]), and declining rates of engagement over time are often reported by researcher-led web-based health interventions.

In a secondary analysis of a randomized trial [42], attrition at 3 months of a 100-day PA intervention delivered via an app ranged from 32% to 39%. Another RCT found that 80% of participants ceased using a web-based PA intervention by week 80 (20 months), and the attrition rate was approximately 70% to 75% at 12 months [43].

The percentage of patients who stopped using the web application and mobile app before 4 months was 32.8% (37/113) in this study.

Thus, the attrition rate observed in our study was consistent with that reported in the literature.

A recent study [44] examined the effect of individualized follow-up with an app for 1 year on peak oxygen uptake in patients undergoing cardiac rehabilitation. The results of this study showed high levels of use of the app in the intervention group: 84% (46/55) of the patients used it to set and achieve personal goals and tasks. The intervention group improved in the peak oxygen uptake to a larger extent than the control group (without the app). Adherence to app use was more than twice the web and app adherence estimated in this study (45/113, 39.8%). This could be mainly explained by the fact that in the study of Lunde et al [44], monitoring and feedback were provided by a real person to the patients, whereas in our study, the PA program was fully automated. The authors explained that the high level of individualization (having a real person behind the app, as well as quite simple technology) may have been crucial to maintaining adherence to app use.

Therefore, adherence in the long term (>10 months) to the web- and mobile-based PA program studied here would be enhanced by introducing engagement with a real PA instructor in the follow-up of the patients.

In our analyses (not shown in the manuscript), we compared the *respondents* and those with *missing* data at 12 months by baseline characteristics.

Those with *missing* data differed from the *respondents* by the baseline declaration of *high PA* and *sitting time during the weekend*. The proportion of those with missing data who declared practicing high PA at baseline was higher than the proportion of the *respondents* (4/58, 6.9% vs 3/170, 1.8%, respectively; *P*=.05). For sitting time, those with *missing* data declared, at baseline, to spend less time sitting during the weekend than the *respondents* (280 vs 320 minutes, respectively; *P*=.046). Nevertheless, those with *missing* data were more frequent in the intervention group than in the control group (36/115, 31.3% vs 22/113, 19.5%, respectively). Therefore, if we hypothesized that those with *missing* data were more active than the *respondents*, the level of PA of the intervention group would have been higher if we had been able to collect data from those with *missing* data.

Finally, in the present analyses, we did not investigate the determinants of which participants were adherent to the program. Such analyses, along with the presentation of the results on the step counts, will be the topic of ongoing analyses.

This clinical trial provided results on the PA of participants attending spa treatment. The generalizability of the results to the general population of older adults with NCDs without spa treatment or rehabilitation programs might be limited. Thus, attending a spa treatment or rehabilitation program is proof of interest in one’s health.

To our knowledge, this study is the first to combine education during a spa treatment and the use of a web- and mobile-based PA program over a 12-month follow-up. The 3-week stay at a spa resort favors the building of strong relations and exchanges with health care professionals and other patients and has an educational dimension [16,20]. This could help explain why various studies have shown that coaching and information on PA administered during spa therapy produces a lasting benefit on PA [17-20] in the intervention groups and also produces an improvement in the controls [20]. Thus, these findings could partly explain why no large differences in PA were observed among patients receiving information in different forms. Moreover, the periodic follow-up by the interviewers in the 2 groups could also be a potential reason for the increasing motivation to practice PA.

### Conclusions

The limitations, especially the impossibility of reaching the required sample size, indicate that it is necessary to interpret the results with caution. Nonetheless, this study demonstrates the potential of a web- and mobile-based PA program associated with an initial face-to-face coaching during a spa treatment to maintain PA in older adults over a 12-month period to achieve PAG and improve quality of life. A spa treatment appears to offer the ideal time and setting to implement education in PA and initiate patients to the use of web- and mobile-based PA programs.

Increasing PA and reducing the excessive sedentariness of inactive patients reduce the risk of NCD aggravation and pain in some nonmalignant chronic conditions, favoring a lasting improvement in personal physical capacity and quality of life.

## Appendix

Multimedia Appendix 1 Use guide of the website.

Multimedia Appendix 2 Use guide of the mobile app.

Multimedia Appendix 3 CONSORT-eHEALTH checklist (V 1.6.1).

## Abbreviations

AE: adverse event
CLDA: constrained longitudinal data analysis
IPAQ: International Physical Activity Questionnaire
M0: month 0
M2: month 2
M6: month 6
M12: month 12
MET: metabolic equivalent of task
NCD: noncommunicable disease
OR: odds ratio
PA: physical activity
PAG: physical activity guidelines
PCS: physical component subscale
RCT: randomized controlled trial
REDCap: Research Electronic Data Capture
SF-12: Short Form Health Survey-12
