# Supplementary material for: Possible Impact of a 12-Month Web- and Smartphone-Based Program to Improve Long-term Physical Activity in Patients Attending Spa Therapy: Randomized Controlled Trial
Source: J Med Internet Res. 2022 Jun 16;24(6):e29640. doi: 10.2196/29640 (PMC9247816; doi:10.2196/29640)
Supplement: Multimedia Appendix 1 [file jmir_v24i6e29640_app1.ppt]

## Slide 1
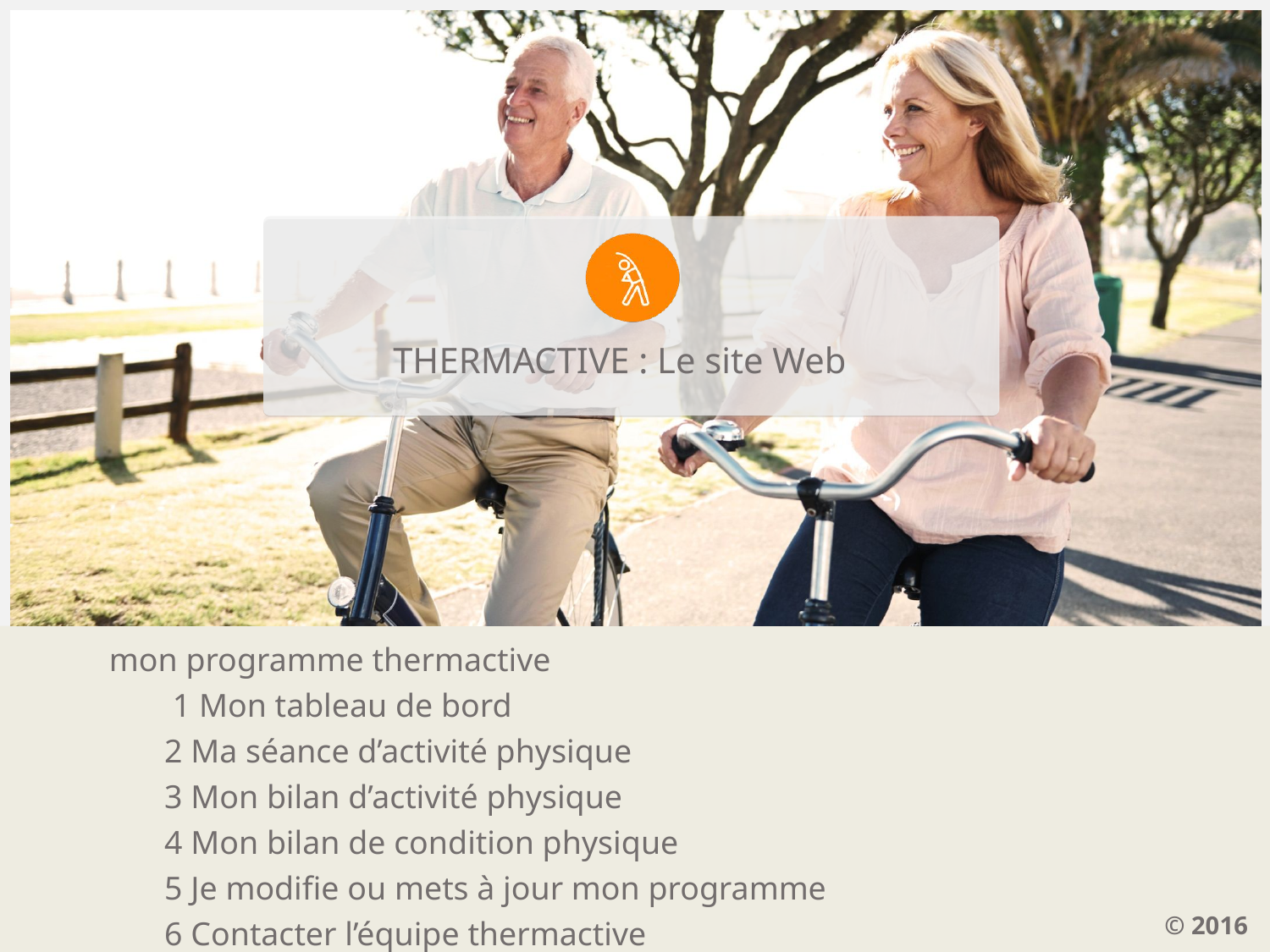

THERMACTIVE : Le site Web
 mon programme thermactive
 1 Mon tableau de bord
2 Ma séance d’activité physique
3 Mon bilan d’activité physique
4 Mon bilan de condition physique
5 Je modifie ou mets à jour mon programme
6 Contacter l’équipe thermactive
© 2016

## Slide 2
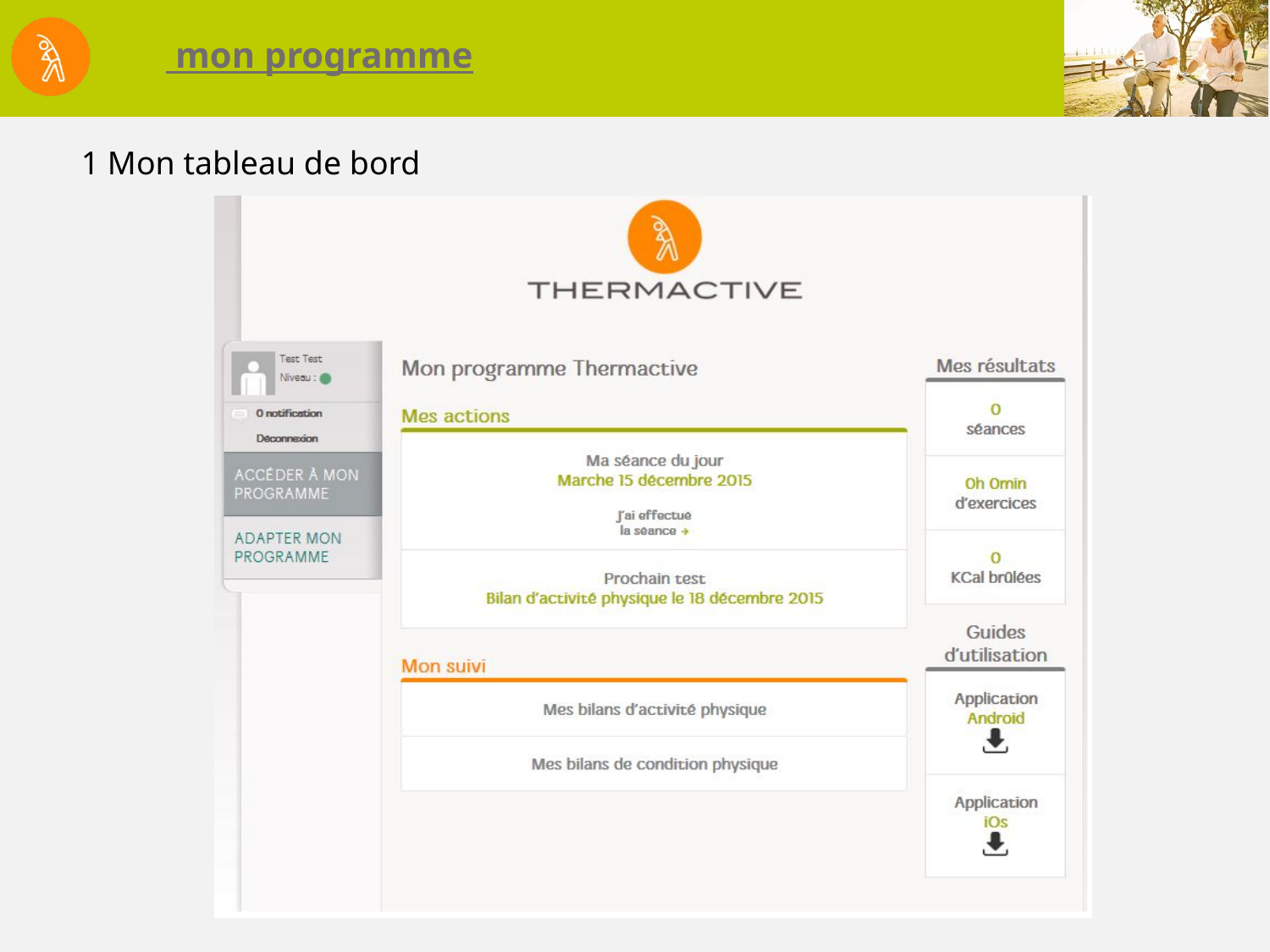

mon programme
 1 Mon tableau de bord

## Slide 3
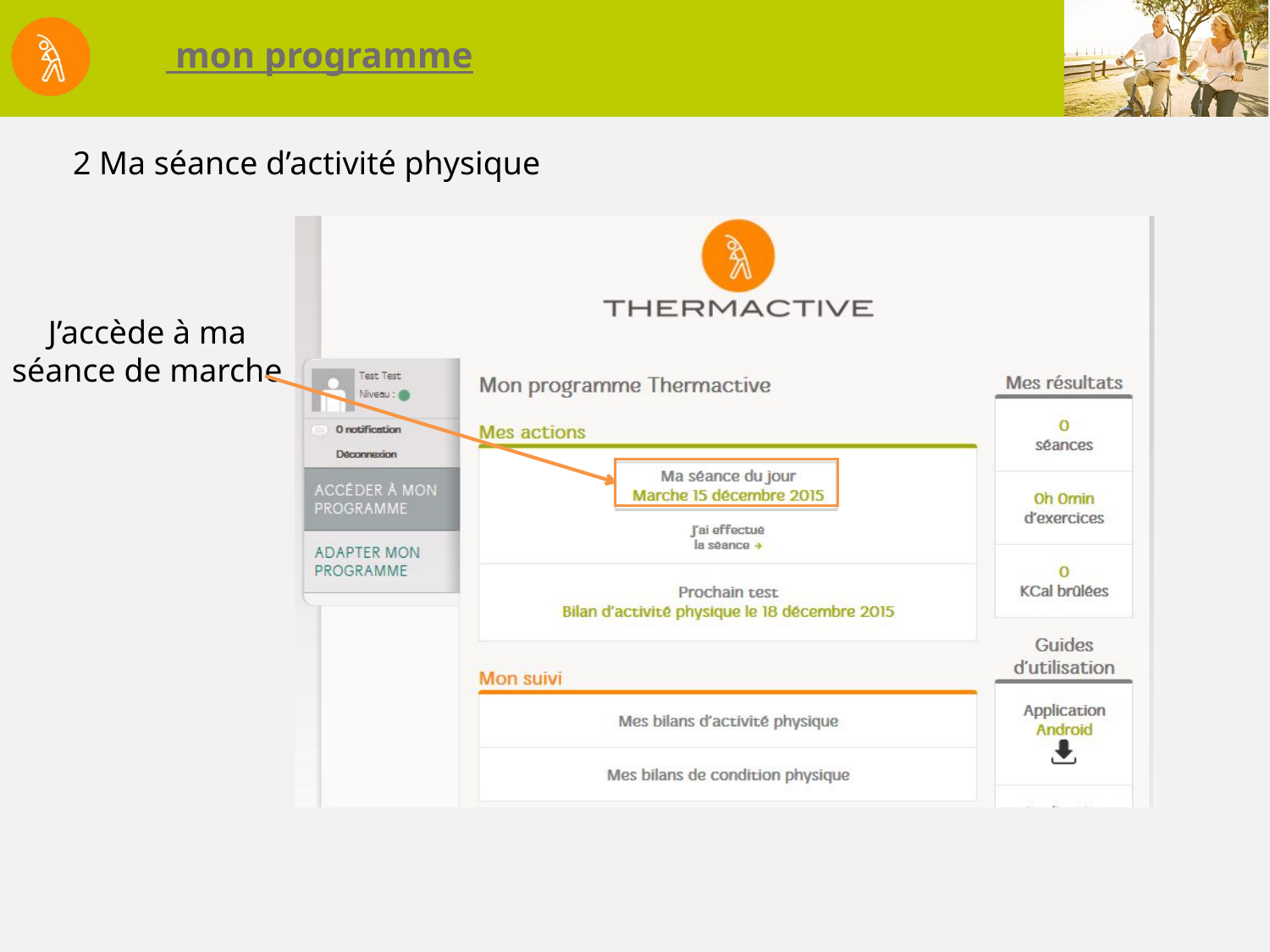

mon programme
2 Ma séance d’activité physique
J’accède à ma séance de marche

## Slide 4
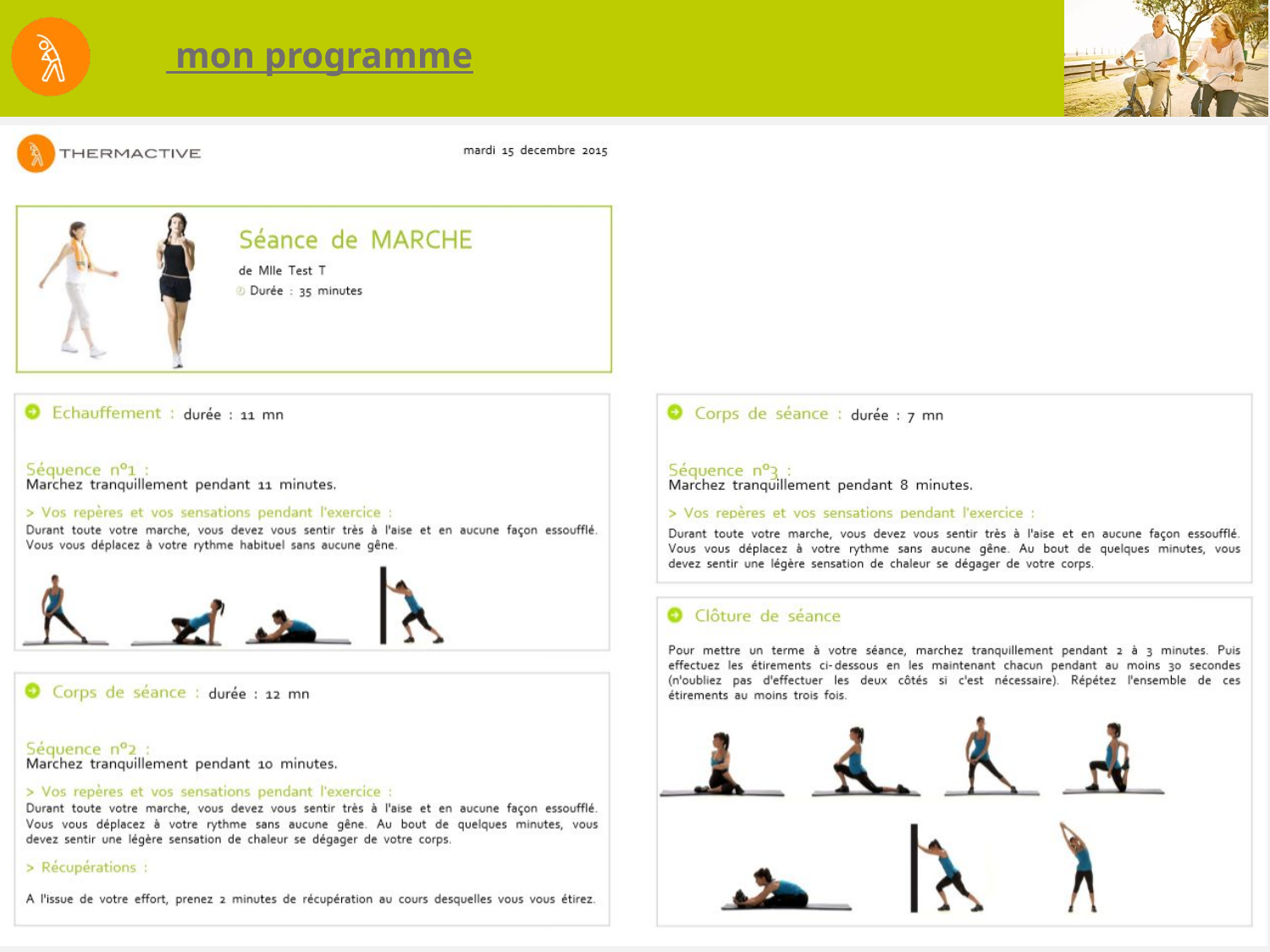

mon programme
 J’accède à ma séance

## Slide 5
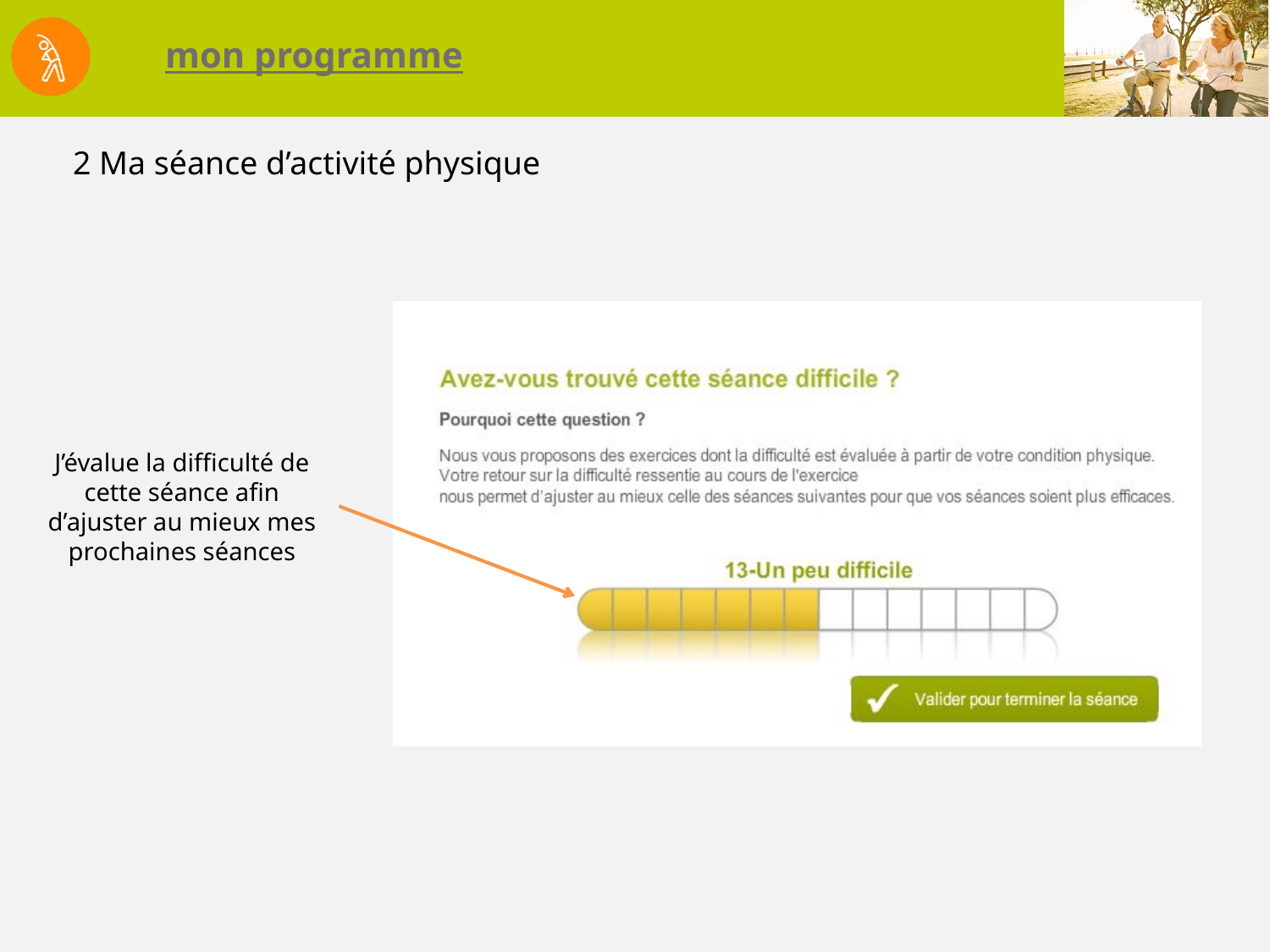

mon programme
2 Ma séance d’activité physique
J’évalue la difficulté de cette séance afin d’ajuster au mieux mes prochaines séances

## Slide 6
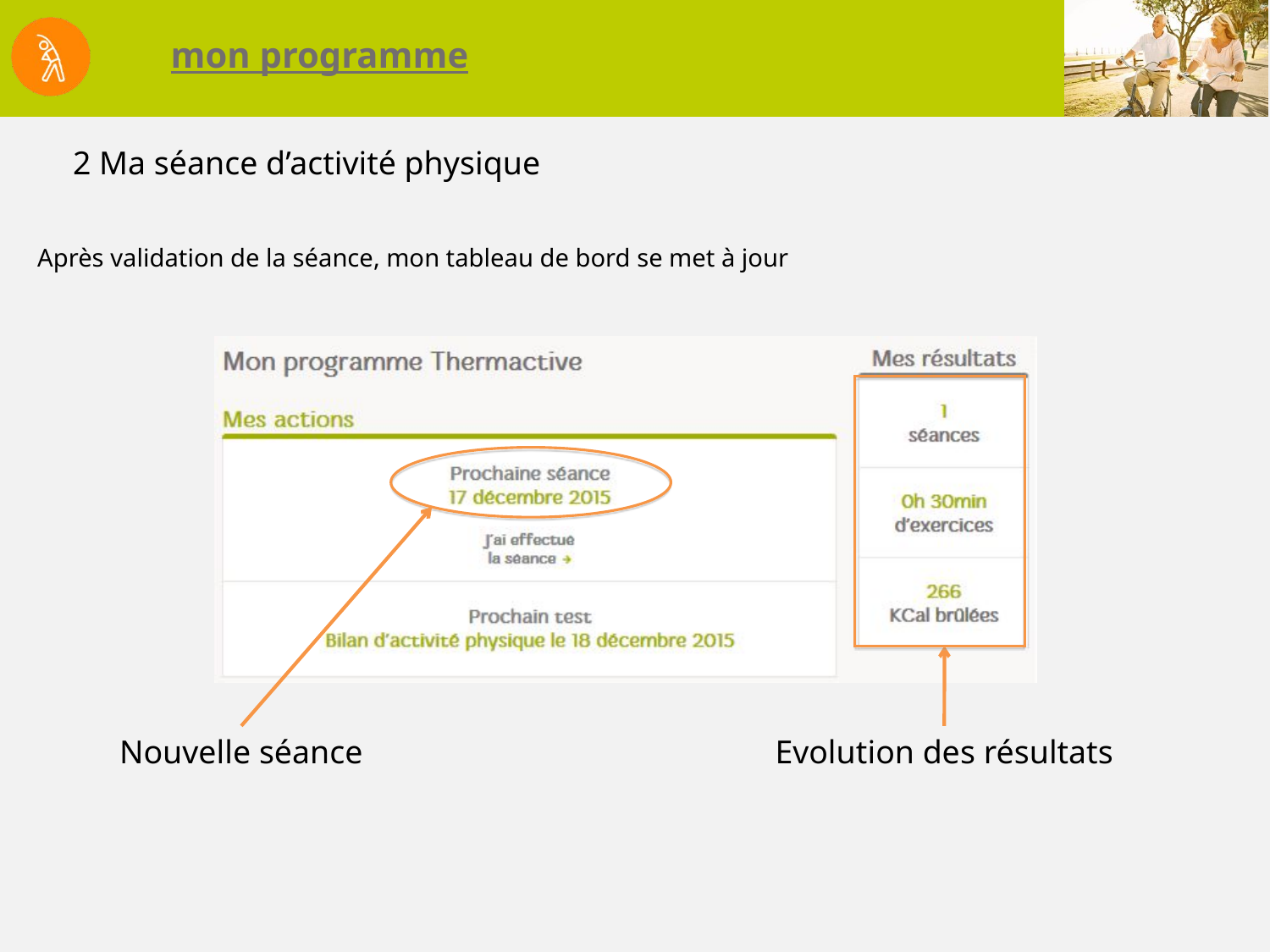

mon programme
2 Ma séance d’activité physique
Après validation de la séance, mon tableau de bord se met à jour
Nouvelle séance
Evolution des résultats

## Slide 7
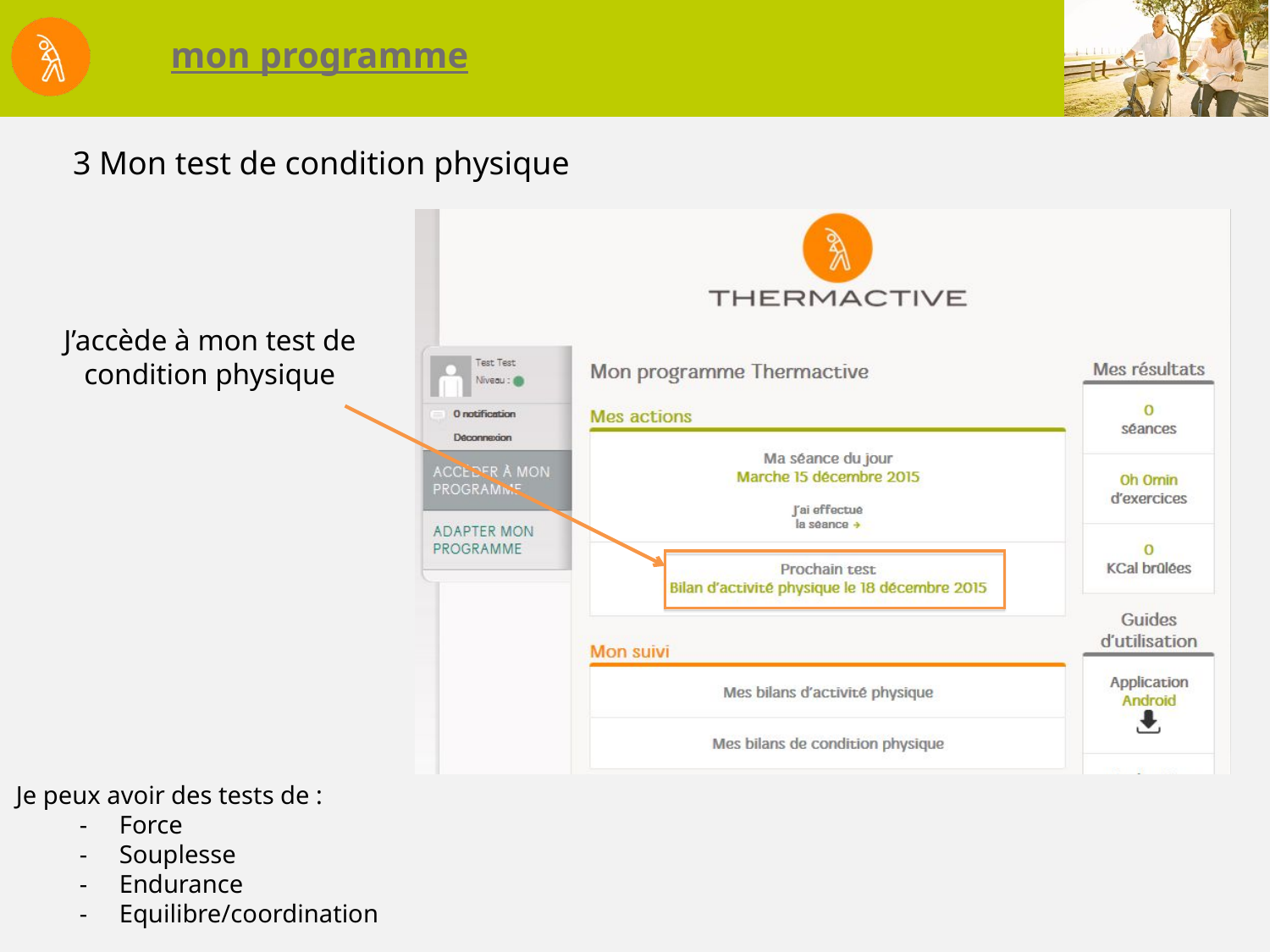

mon programme
3 Mon test de condition physique
J’accède à mon test de condition physique
Je peux avoir des tests de :
Force
Souplesse
Endurance
Equilibre/coordination

## Slide 8
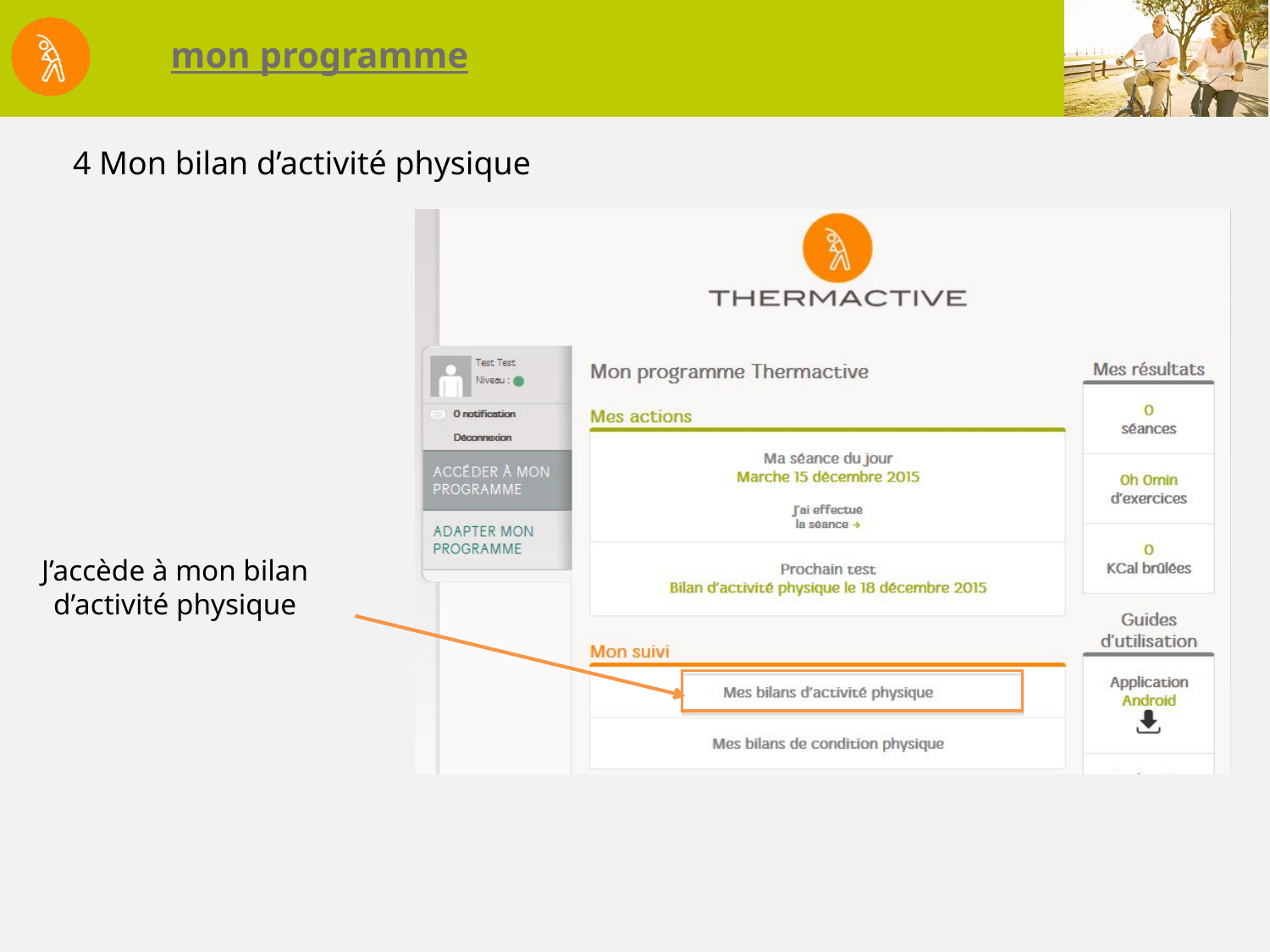

mon programme
4 Mon bilan d’activité physique
J’accède à mon bilan d’activité physique

## Slide 9
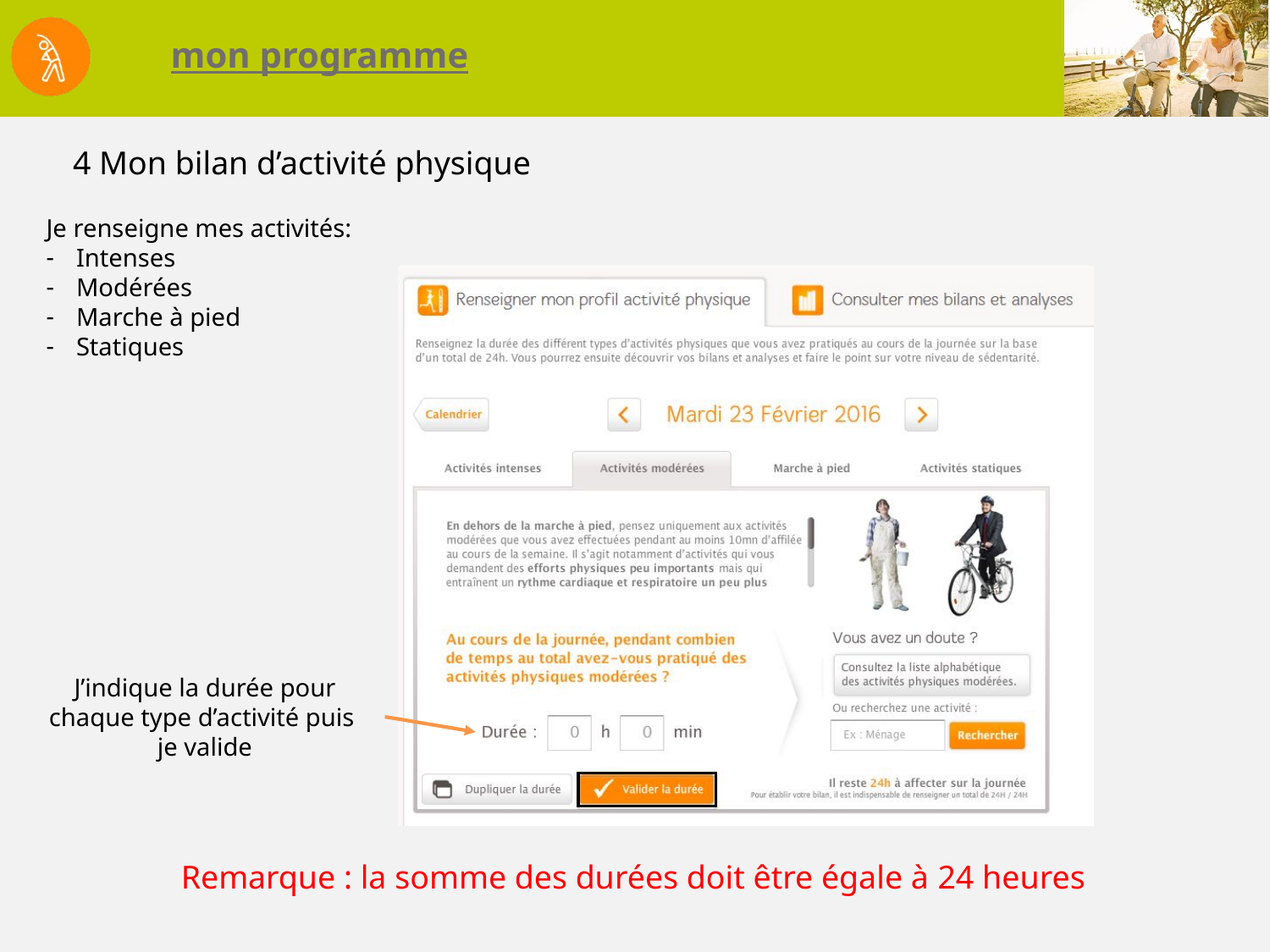

mon programme
4 Mon bilan d’activité physique
Je renseigne mes activités:
Intenses
Modérées
Marche à pied
Statiques
J’indique la durée pour chaque type d’activité puis
je valide
Remarque : la somme des durées doit être égale à 24 heures

## Slide 10
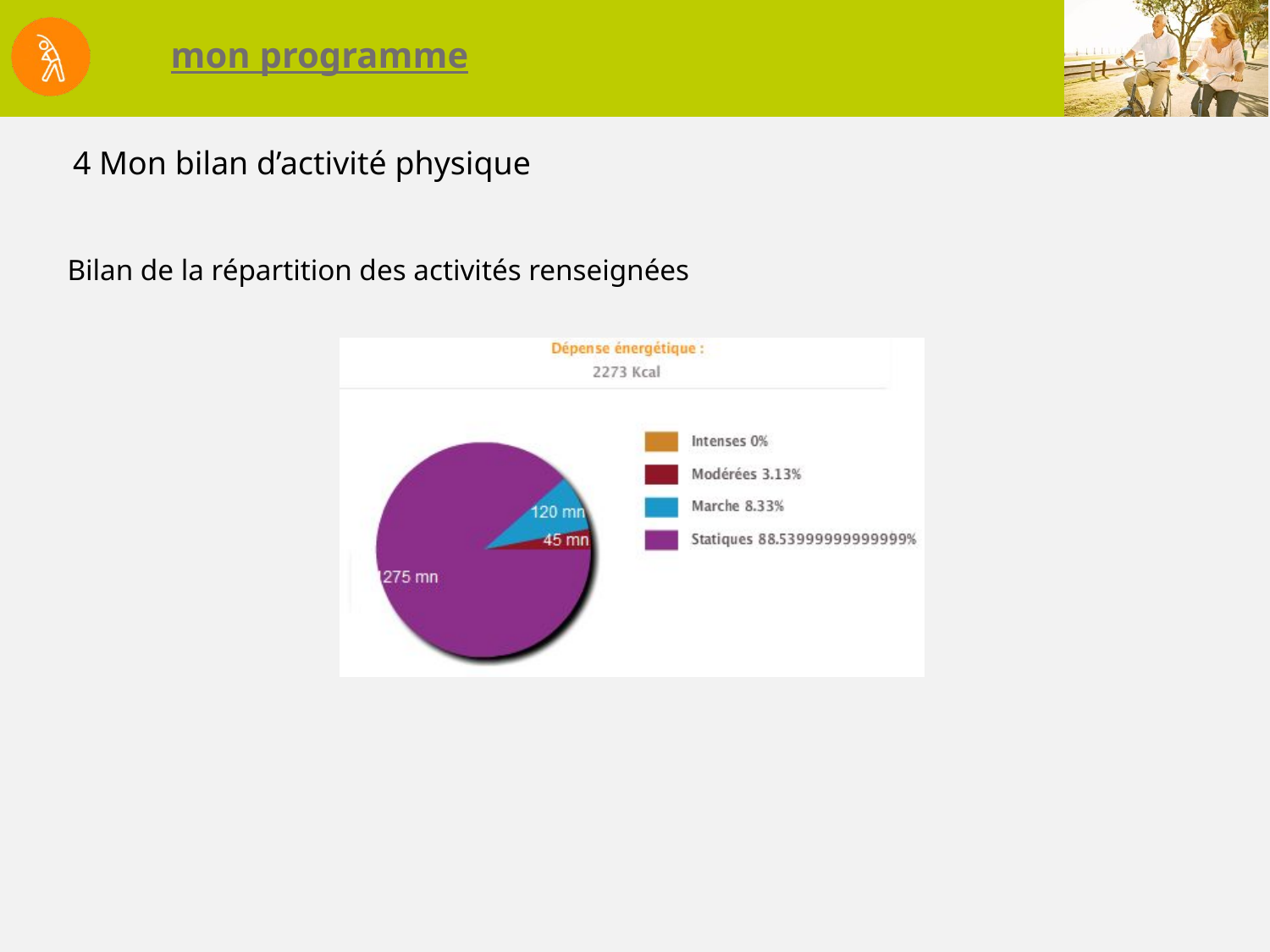

mon programme
4 Mon bilan d’activité physique
Bilan de la répartition des activités renseignées

## Slide 11
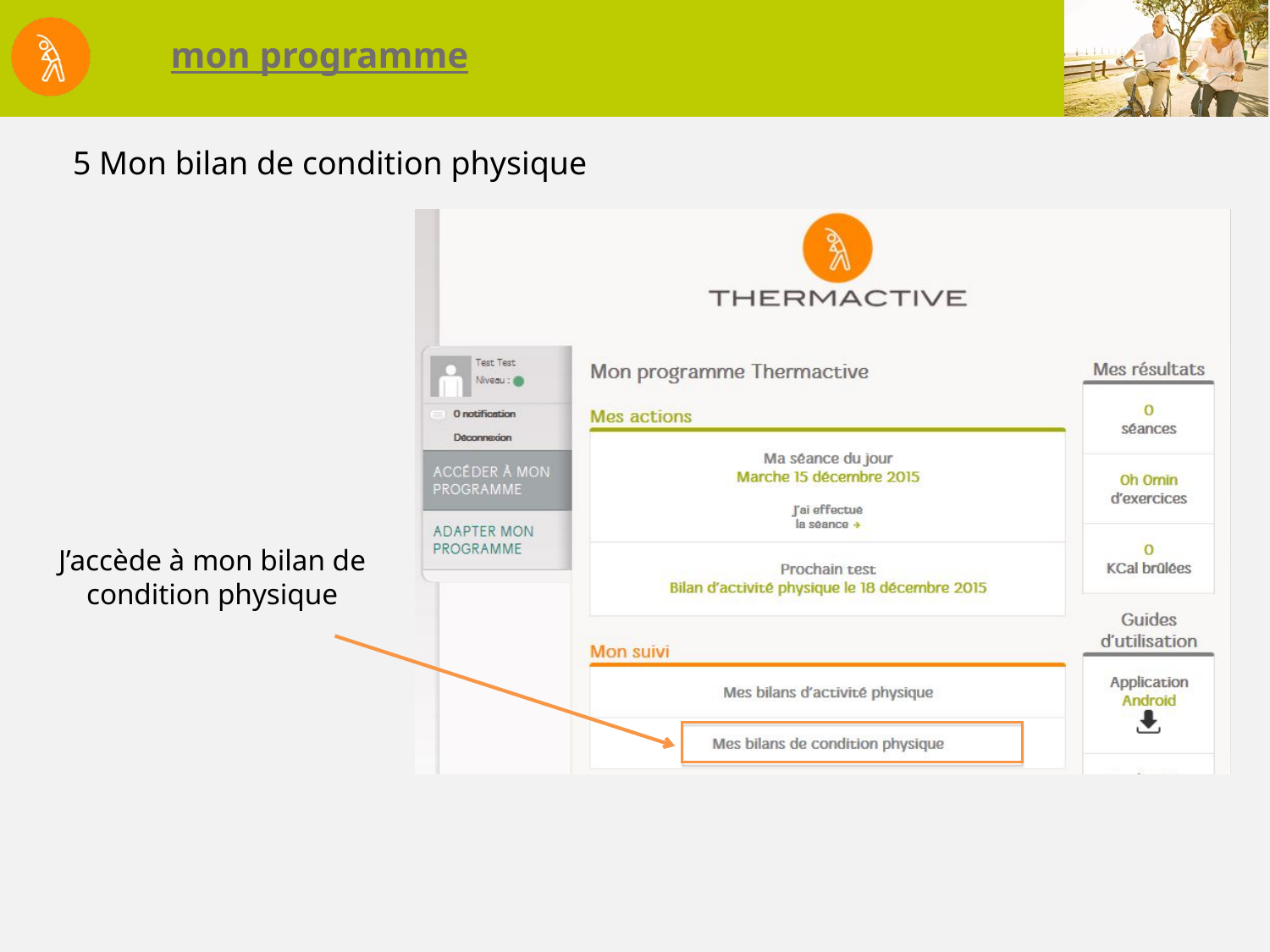

mon programme
5 Mon bilan de condition physique
J’accède à mon bilan de condition physique

## Slide 12
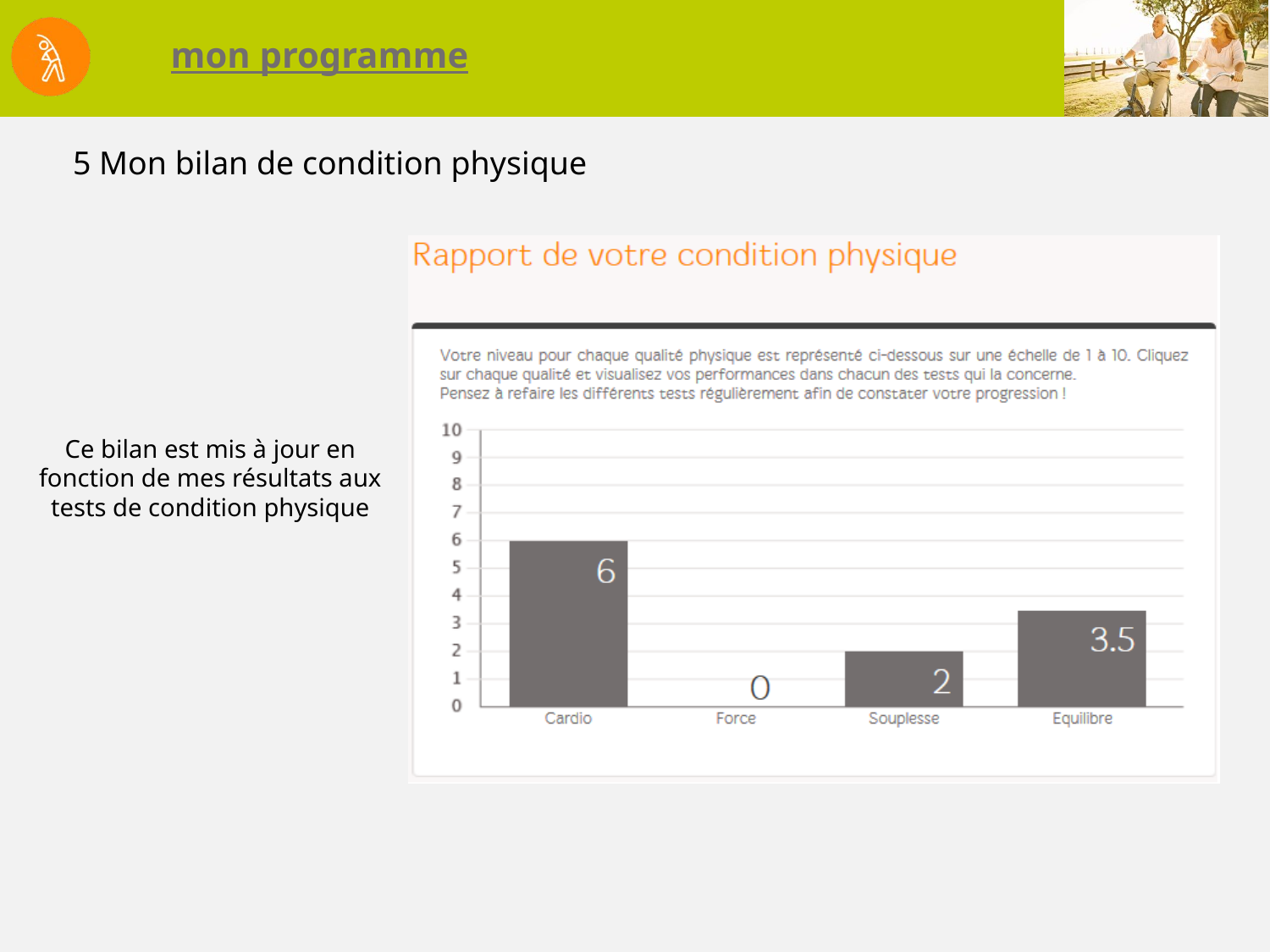

mon programme
5 Mon bilan de condition physique
Ce bilan est mis à jour en fonction de mes résultats aux tests de condition physique

## Slide 13
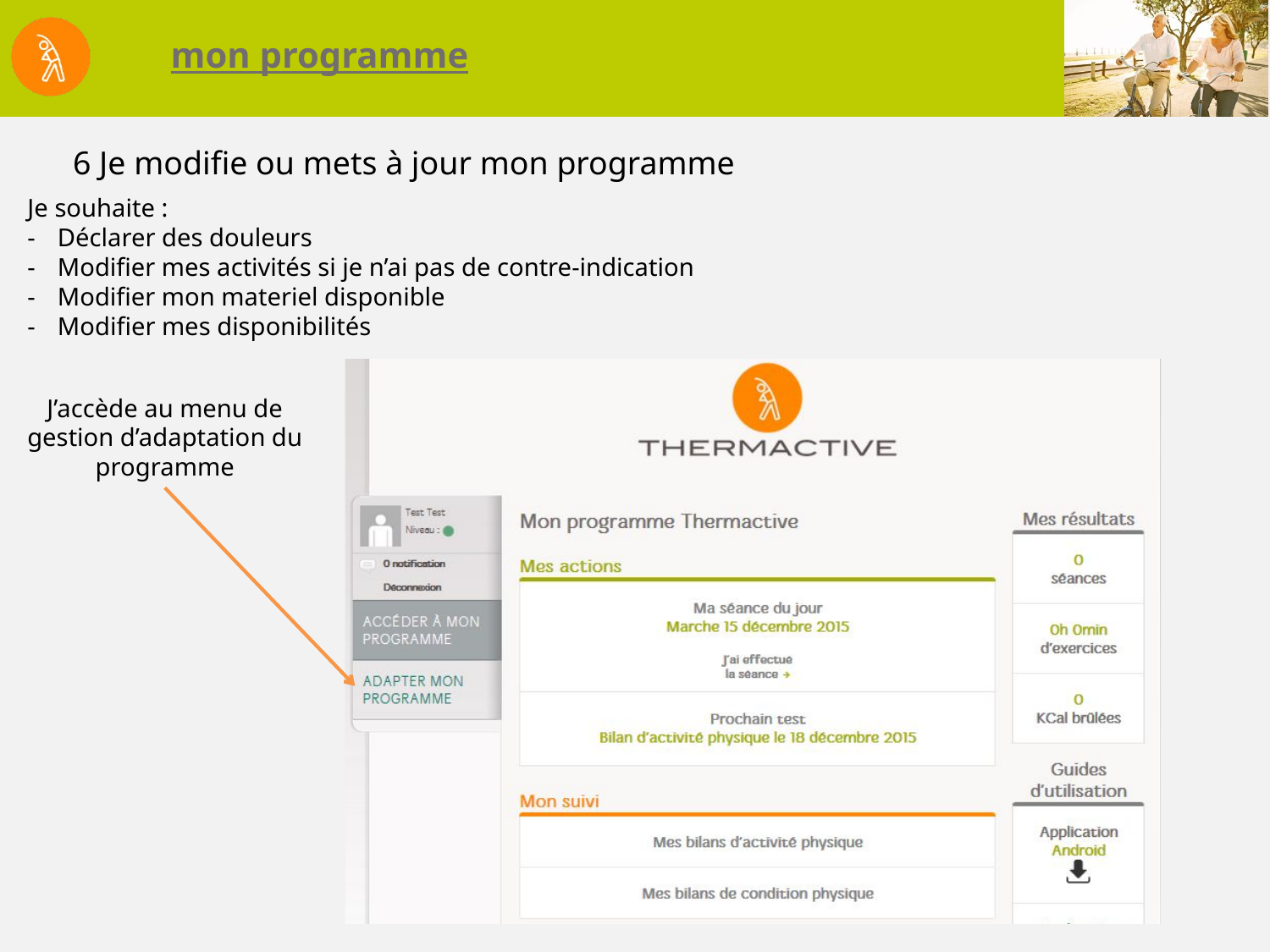

mon programme
6 Je modifie ou mets à jour mon programme
Je souhaite :
Déclarer des douleurs
Modifier mes activités si je n’ai pas de contre-indication
Modifier mon materiel disponible
Modifier mes disponibilités
J’accède au menu de gestion d’adaptation du programme

## Slide 14
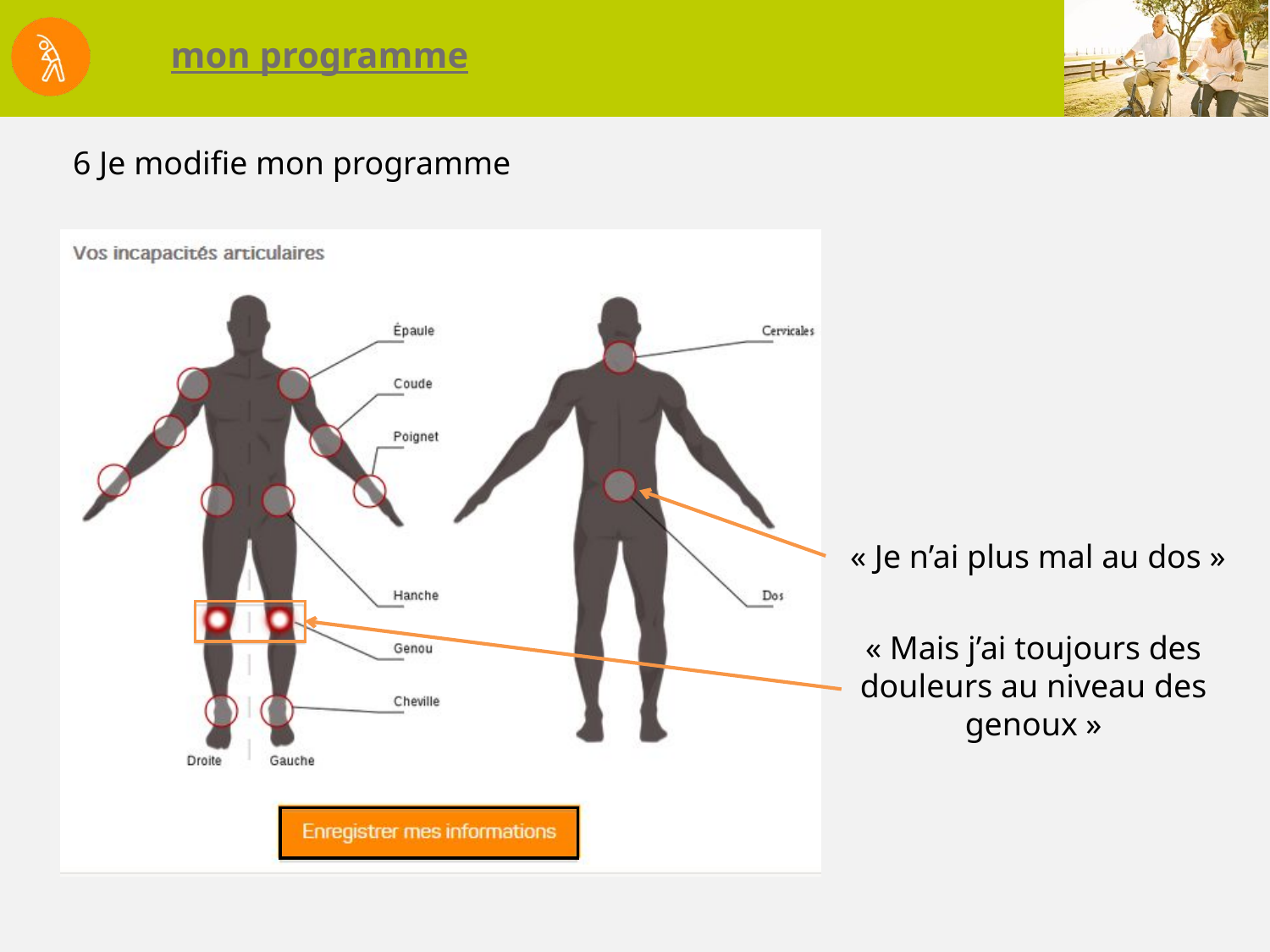

mon programme
6 Je modifie mon programme
« Je n’ai plus mal au dos »
« Mais j’ai toujours des douleurs au niveau des genoux »

## Slide 15
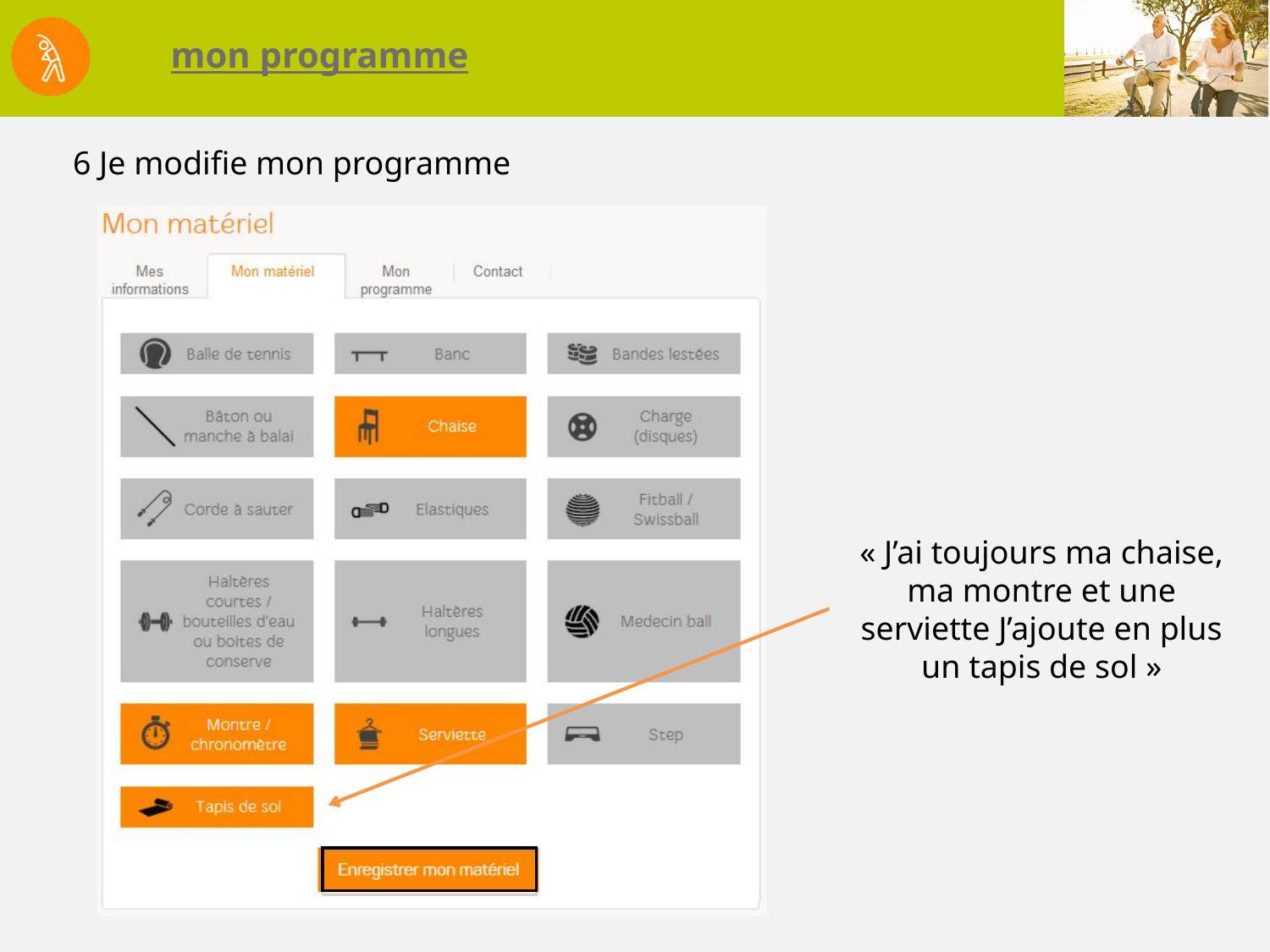

mon programme
6 Je modifie mon programme
« J’ai toujours ma chaise, ma montre et une serviette J’ajoute en plus un tapis de sol »

## Slide 16
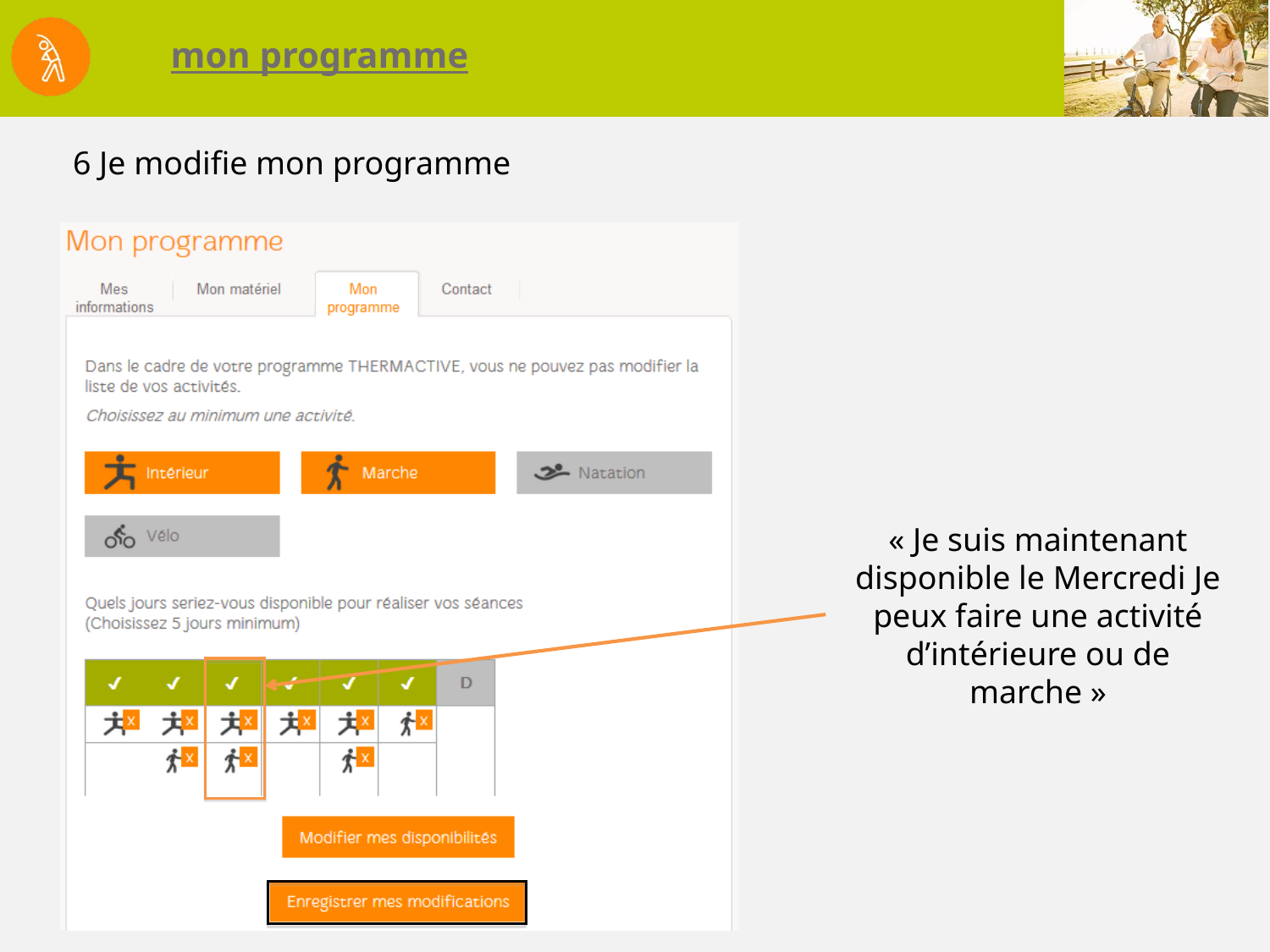

mon programme
6 Je modifie mon programme
« Je suis maintenant disponible le Mercredi Je peux faire une activité d’intérieure ou de marche »

## Slide 17
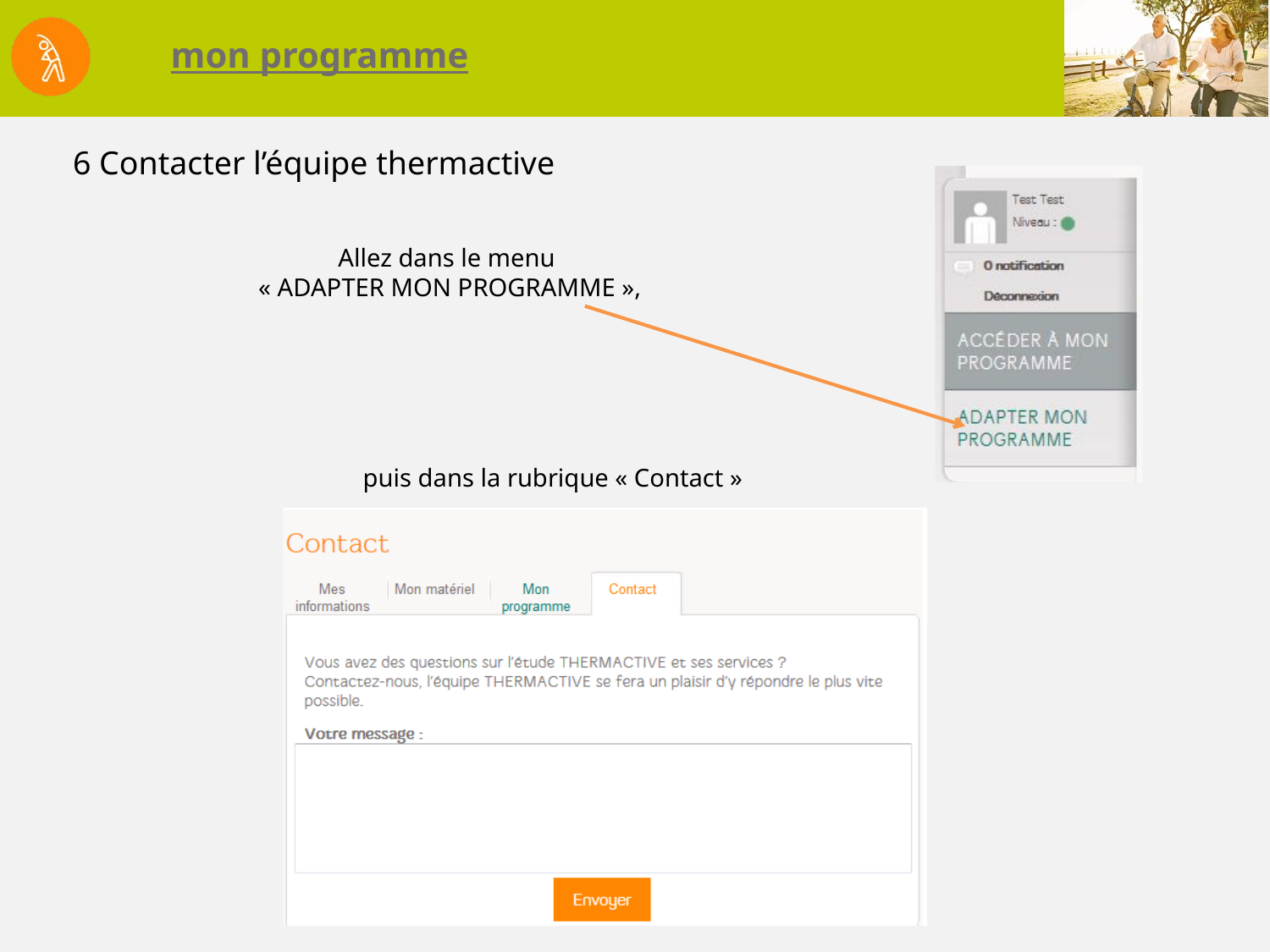

mon programme
6 Contacter l’équipe thermactive
Allez dans le menu
« ADAPTER MON PROGRAMME »,
puis dans la rubrique « Contact »
