# Supplementary material for: Possible Impact of a 12-Month Web- and Smartphone-Based Program to Improve Long-term Physical Activity in Patients Attending Spa Therapy: Randomized Controlled Trial
Source: J Med Internet Res. 2022 Jun 16;24(6):e29640. doi: 10.2196/29640 (PMC9247816; doi:10.2196/29640)
Supplement: Multimedia Appendix 2 [file jmir_v24i6e29640_app2.ppt]

## Slide 1
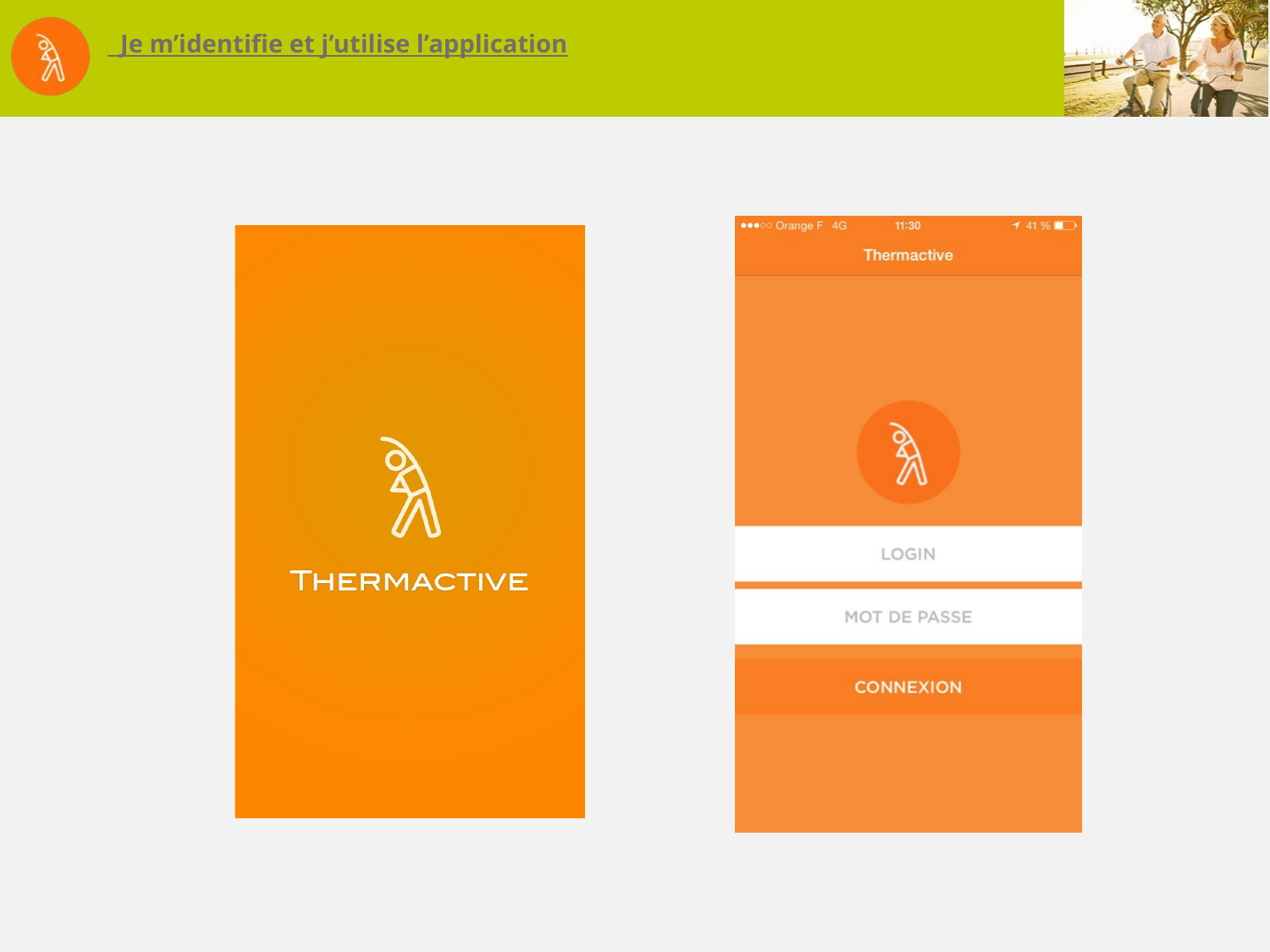

Je m’identifie et j’utilise l’application

## Slide 2
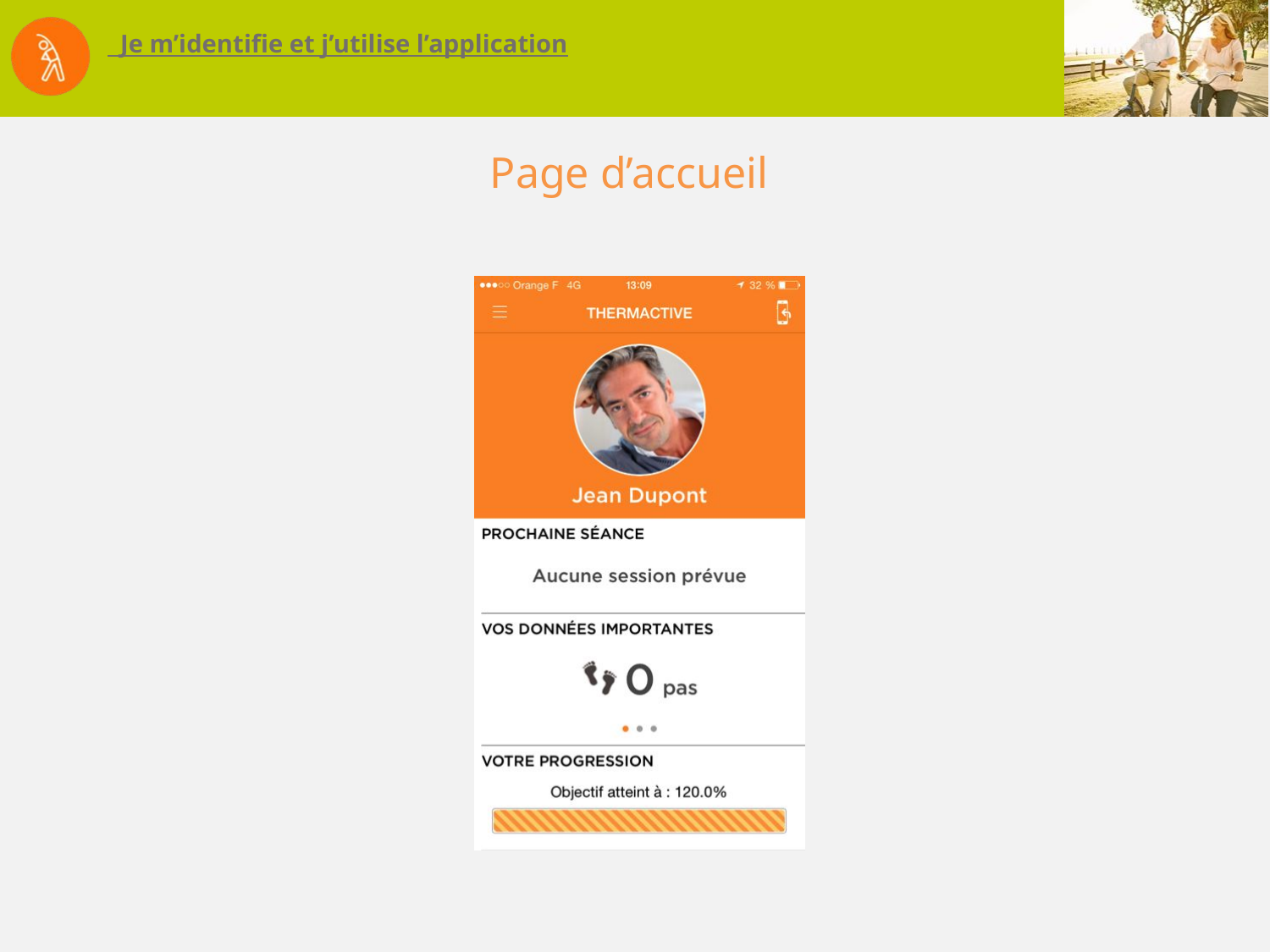

Je m’identifie et j’utilise l’application
Page d’accueil

## Slide 3
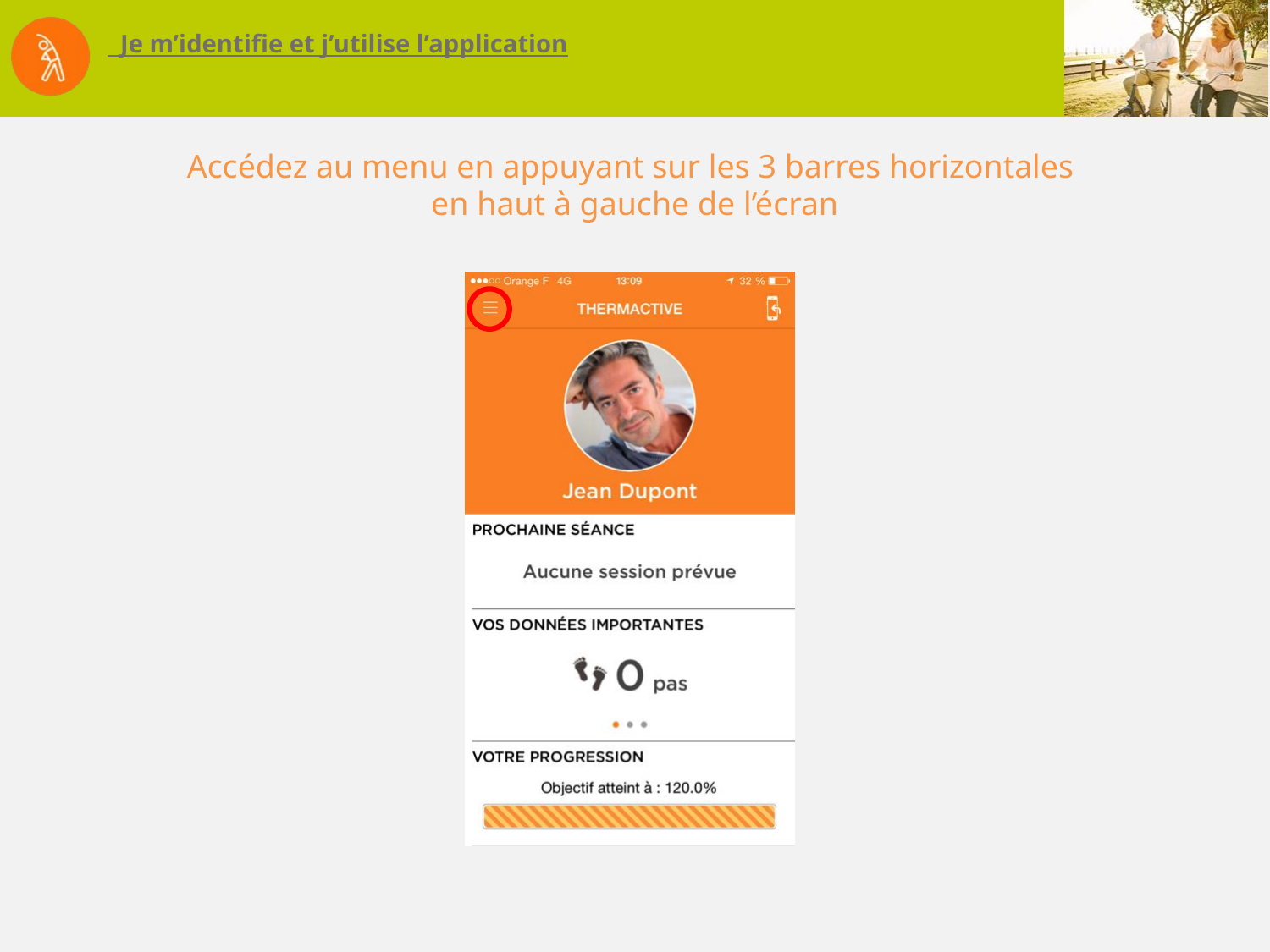

Je m’identifie et j’utilise l’application
Accédez au menu en appuyant sur les 3 barres horizontales
en haut à gauche de l’écran

## Slide 4
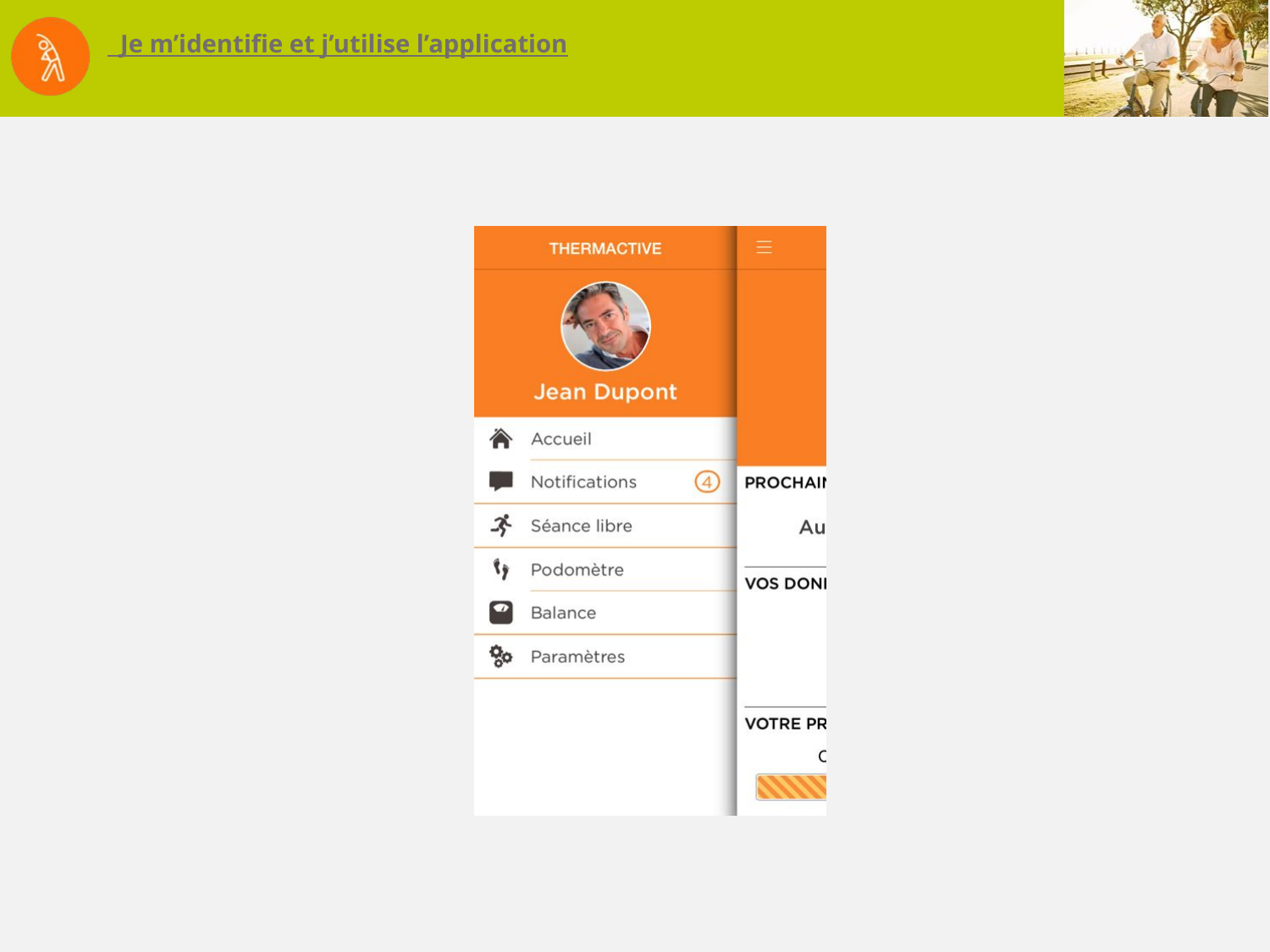

Je m’identifie et j’utilise l’application

## Slide 5
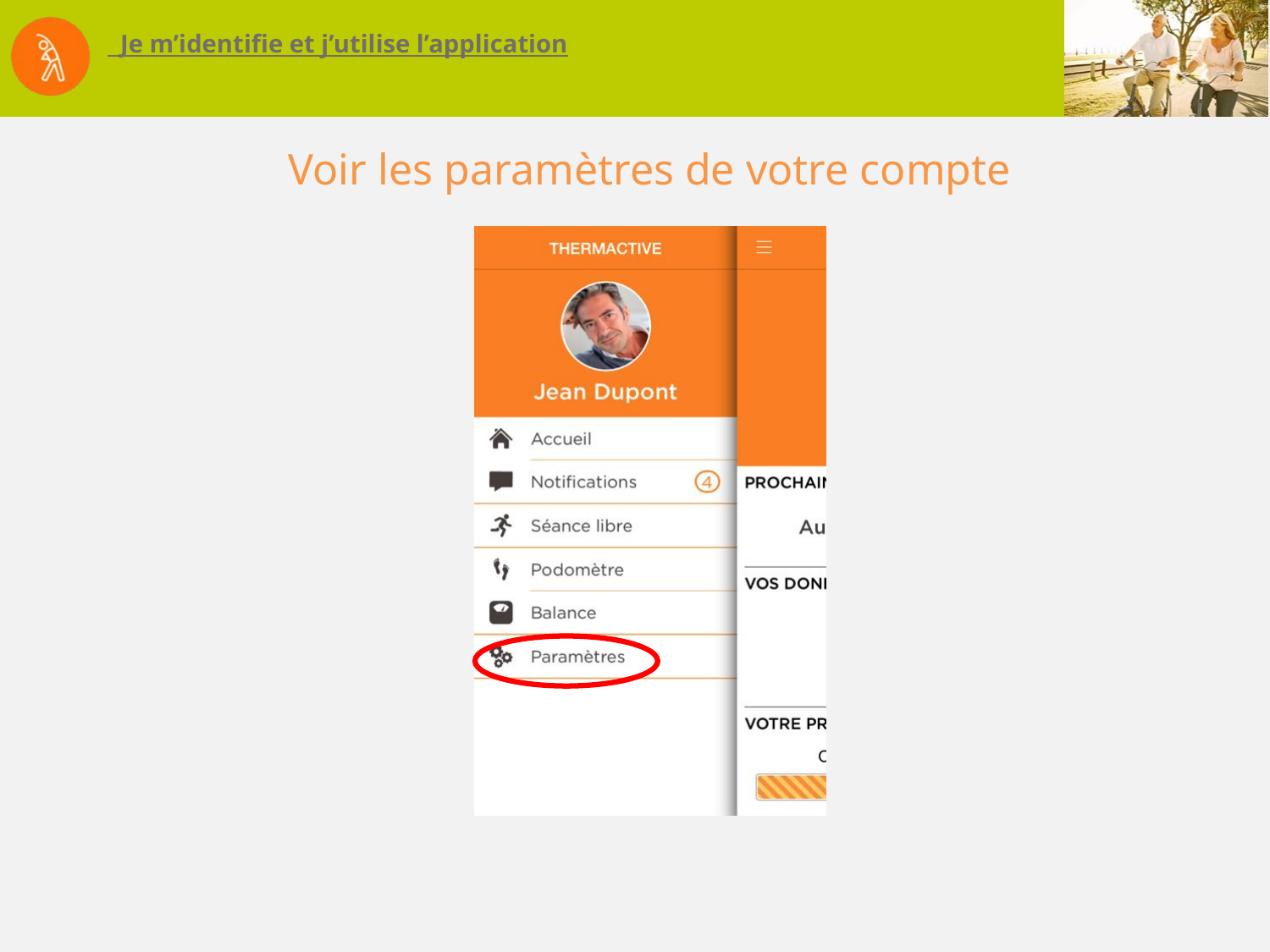

Je m’identifie et j’utilise l’application
Voir les paramètres de votre compte

## Slide 6
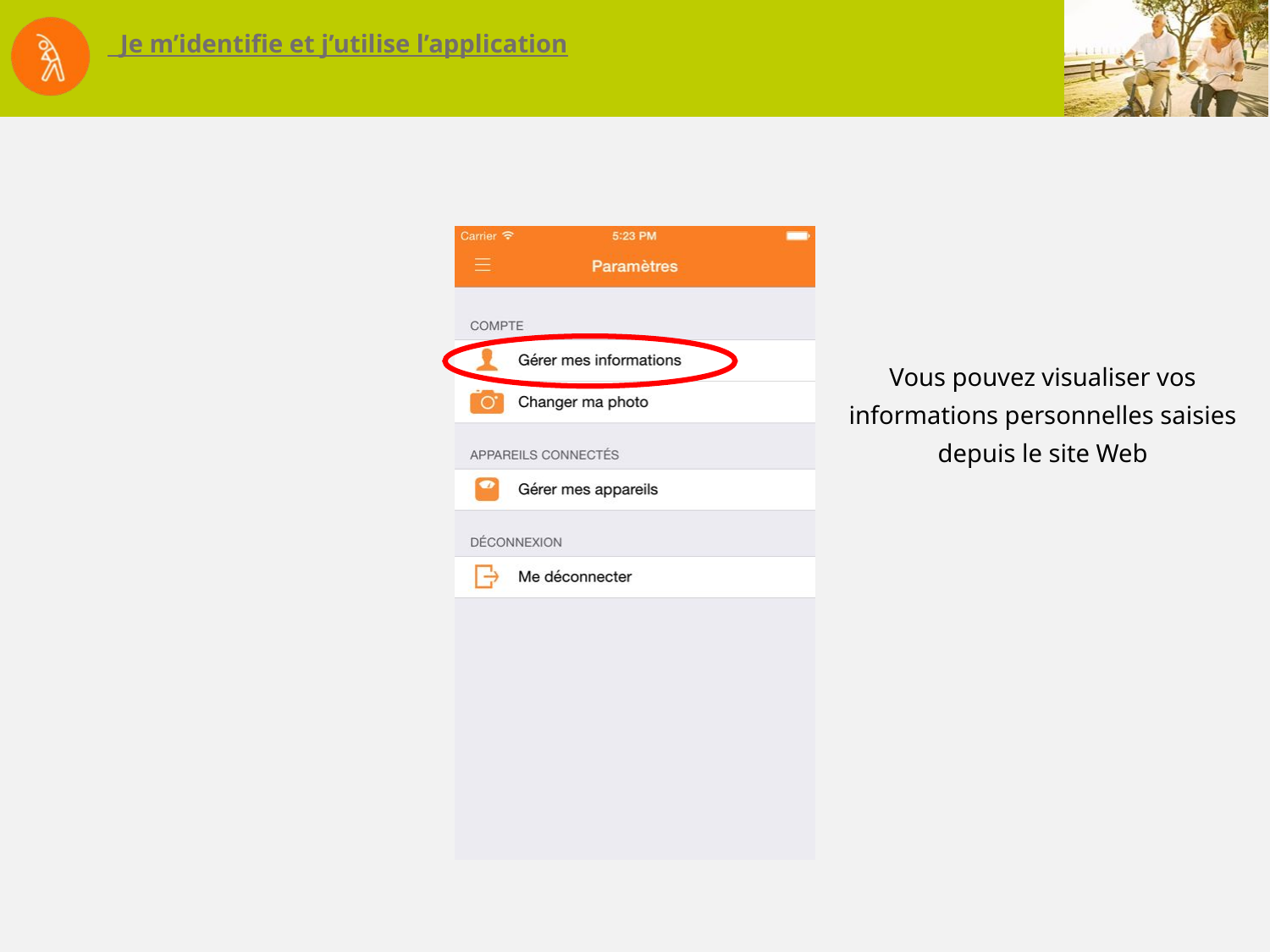

Je m’identifie et j’utilise l’application
Vous pouvez visualiser vos informations personnelles saisies depuis le site Web

## Slide 7
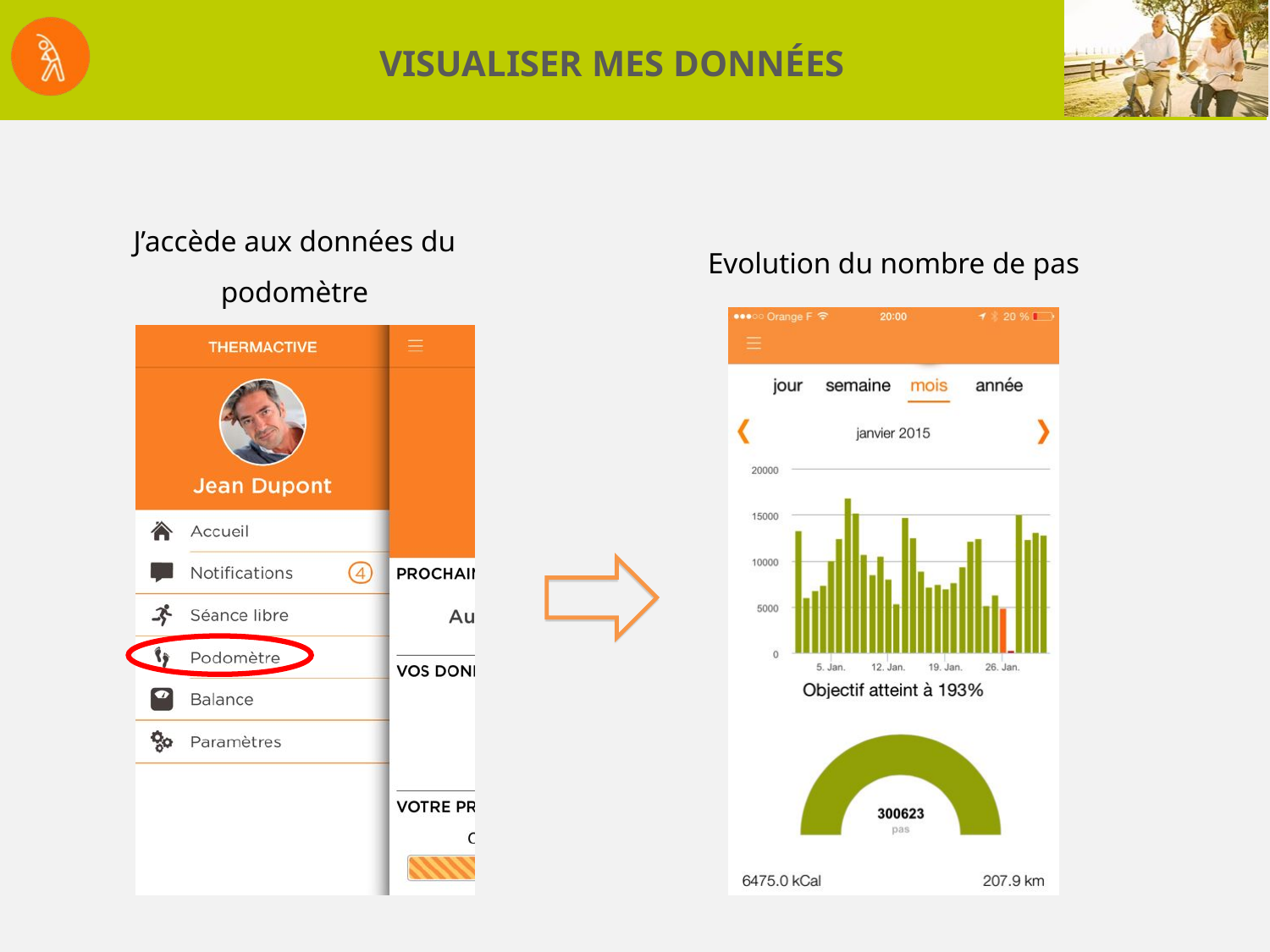

VISUALISER MES DONNÉES
J’accède aux données du podomètre
Evolution du nombre de pas

## Slide 8
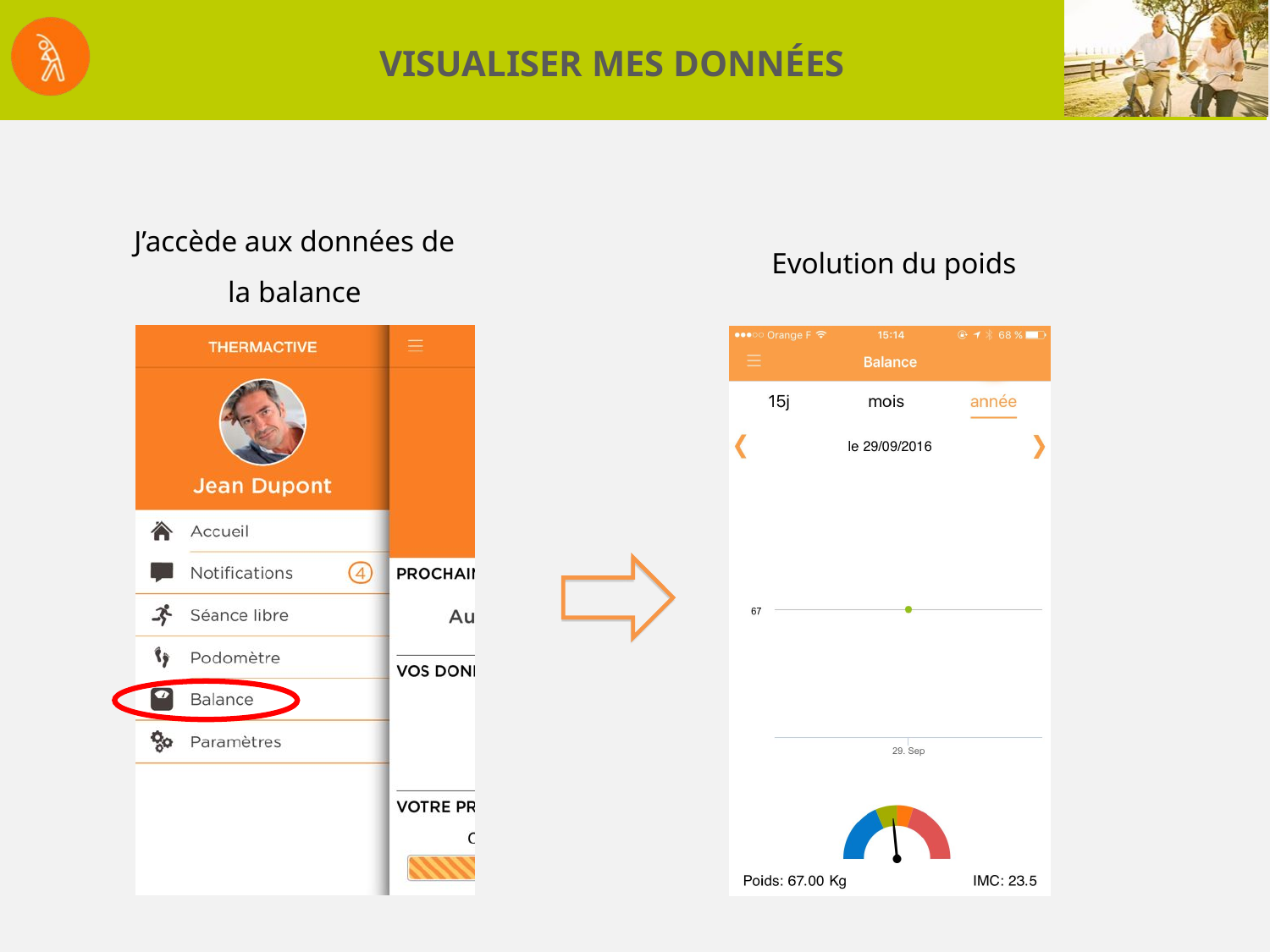

VISUALISER MES DONNÉES
J’accède aux données de la balance
Evolution du poids

## Slide 9
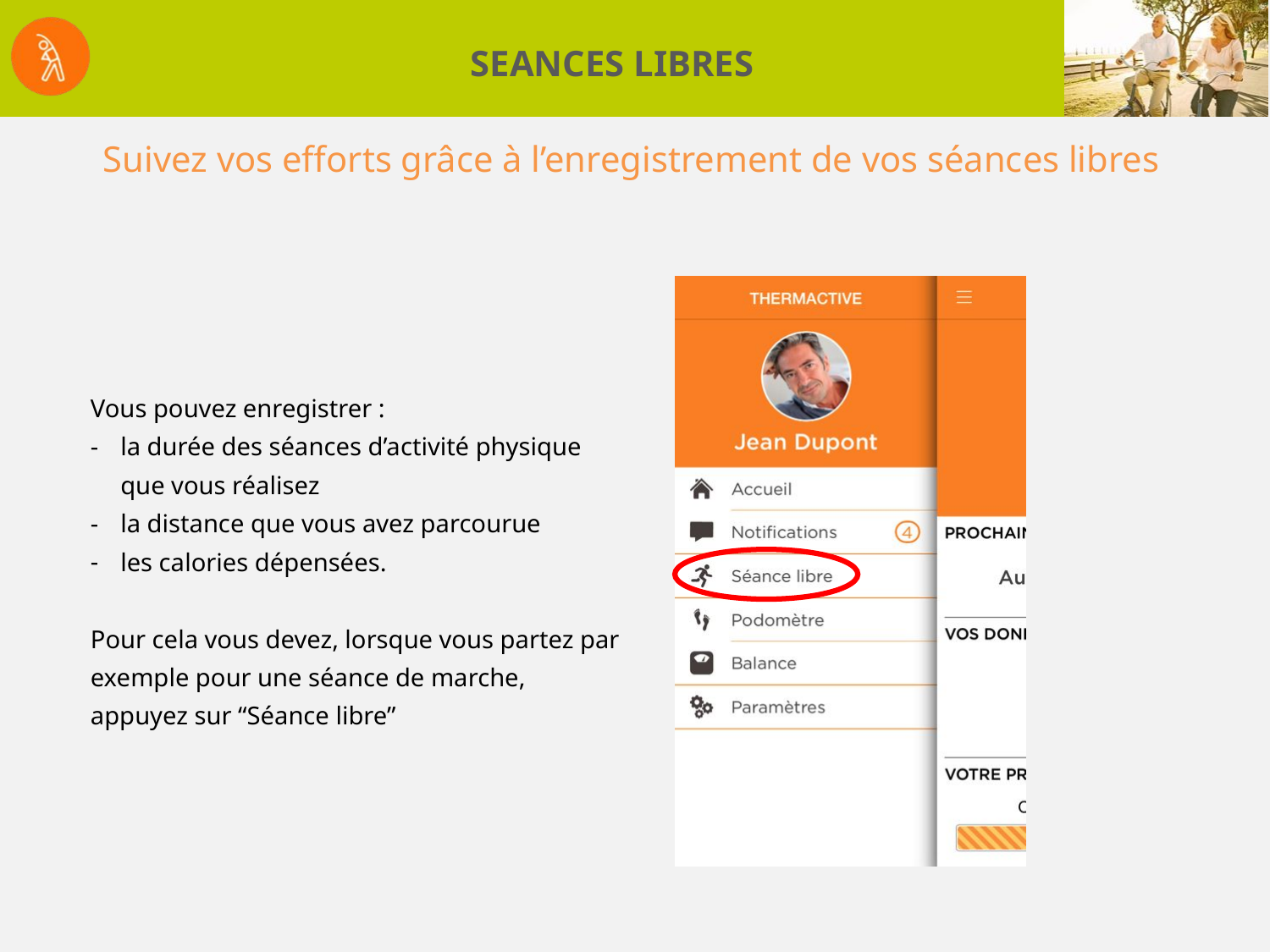

SEANCES LIBRES
Suivez vos efforts grâce à l’enregistrement de vos séances libres
Vous pouvez enregistrer :
la durée des séances d’activité physique que vous réalisez
la distance que vous avez parcourue
les calories dépensées.
Pour cela vous devez, lorsque vous partez par exemple pour une séance de marche, appuyez sur “Séance libre”

## Slide 10
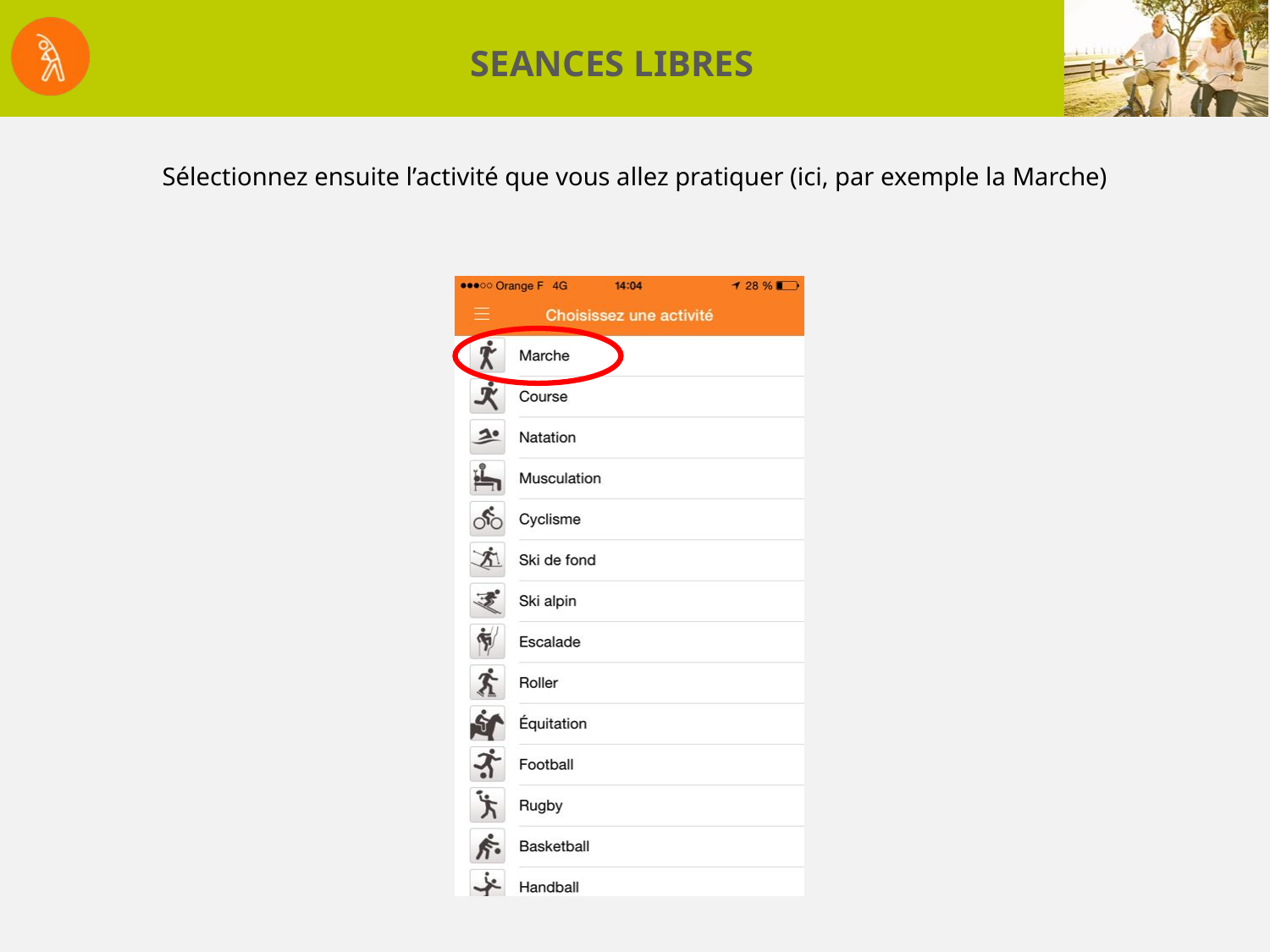

SEANCES LIBRES
Sélectionnez ensuite l’activité que vous allez pratiquer (ici, par exemple la Marche)

## Slide 11
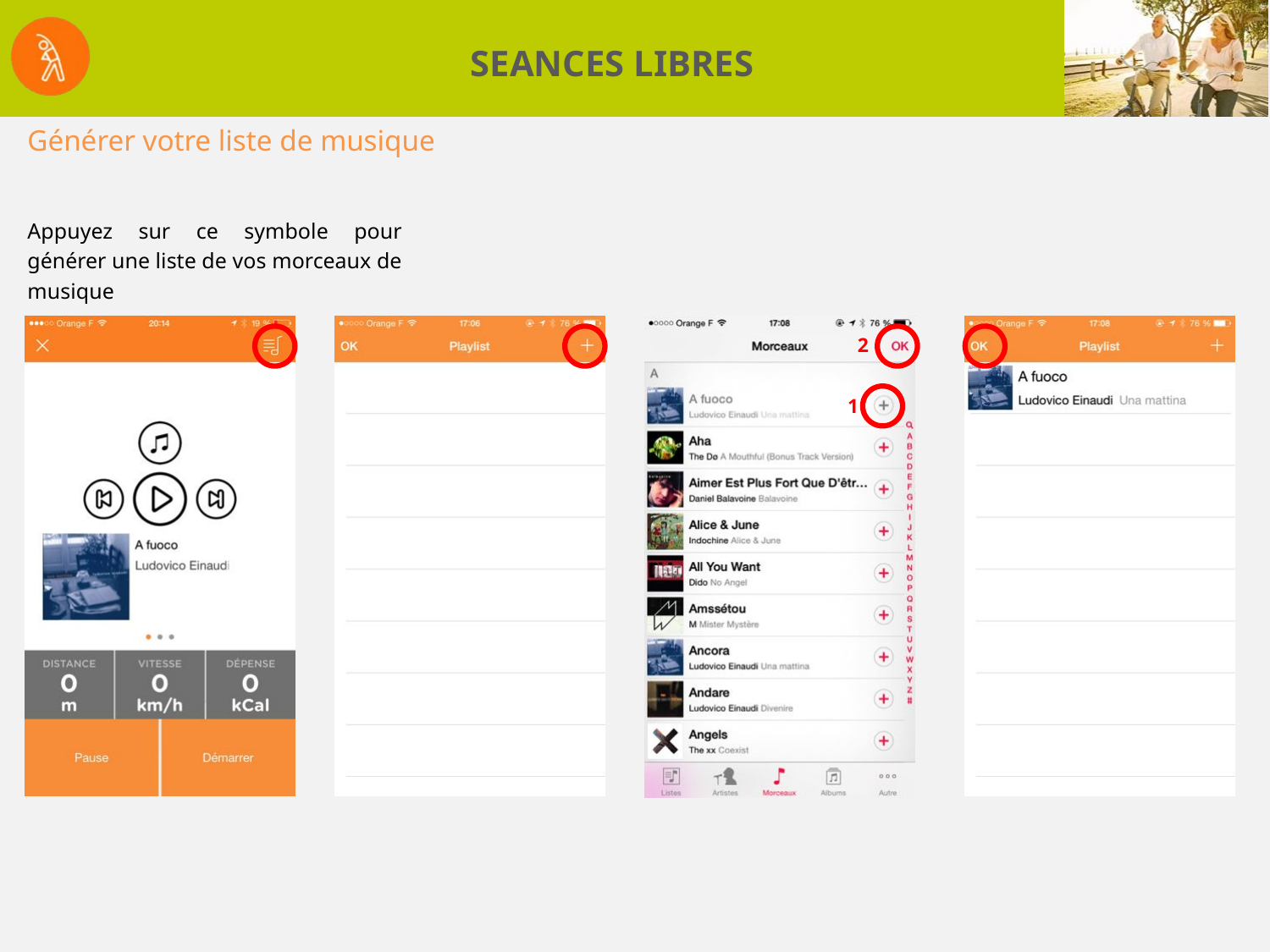

SEANCES LIBRES
Générer votre liste de musique
Appuyez sur ce symbole pour générer une liste de vos morceaux de musique
2
1

## Slide 12
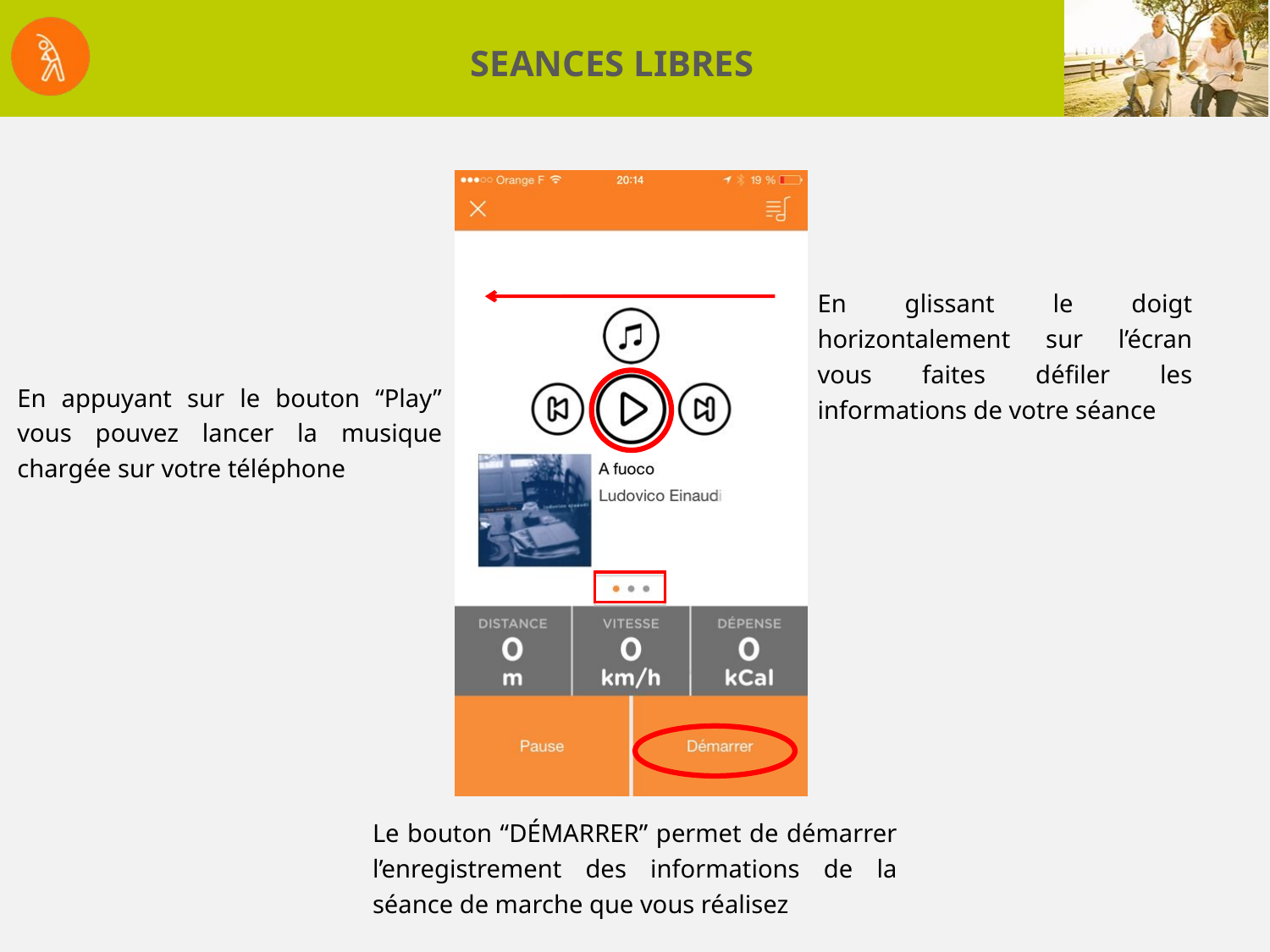

SEANCES LIBRES
En glissant le doigt horizontalement sur l’écran vous faites défiler les informations de votre séance
En appuyant sur le bouton “Play” vous pouvez lancer la musique chargée sur votre téléphone
Le bouton “DÉMARRER” permet de démarrer l’enregistrement des informations de la séance de marche que vous réalisez

## Slide 13
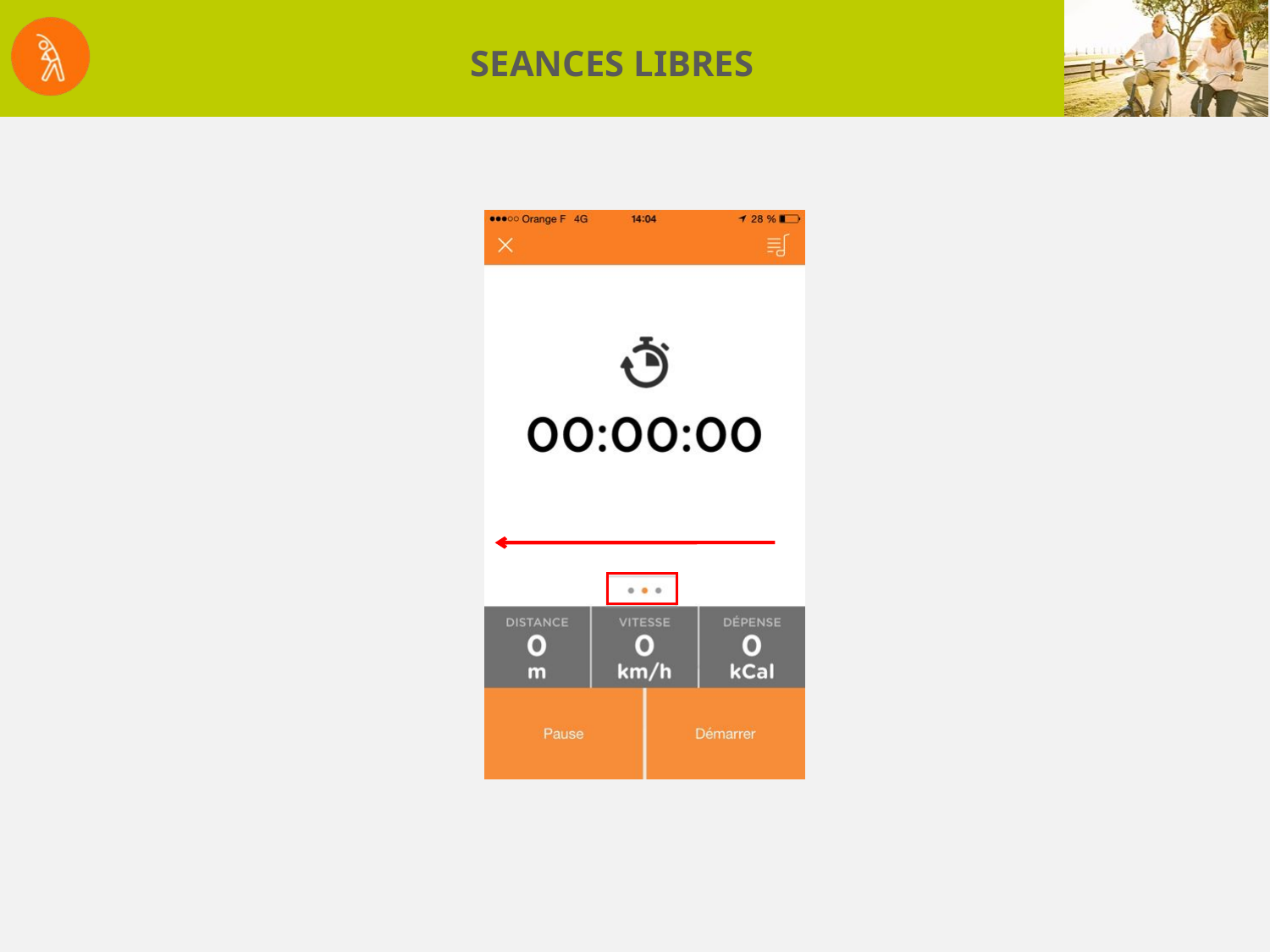

SEANCES LIBRES

## Slide 14
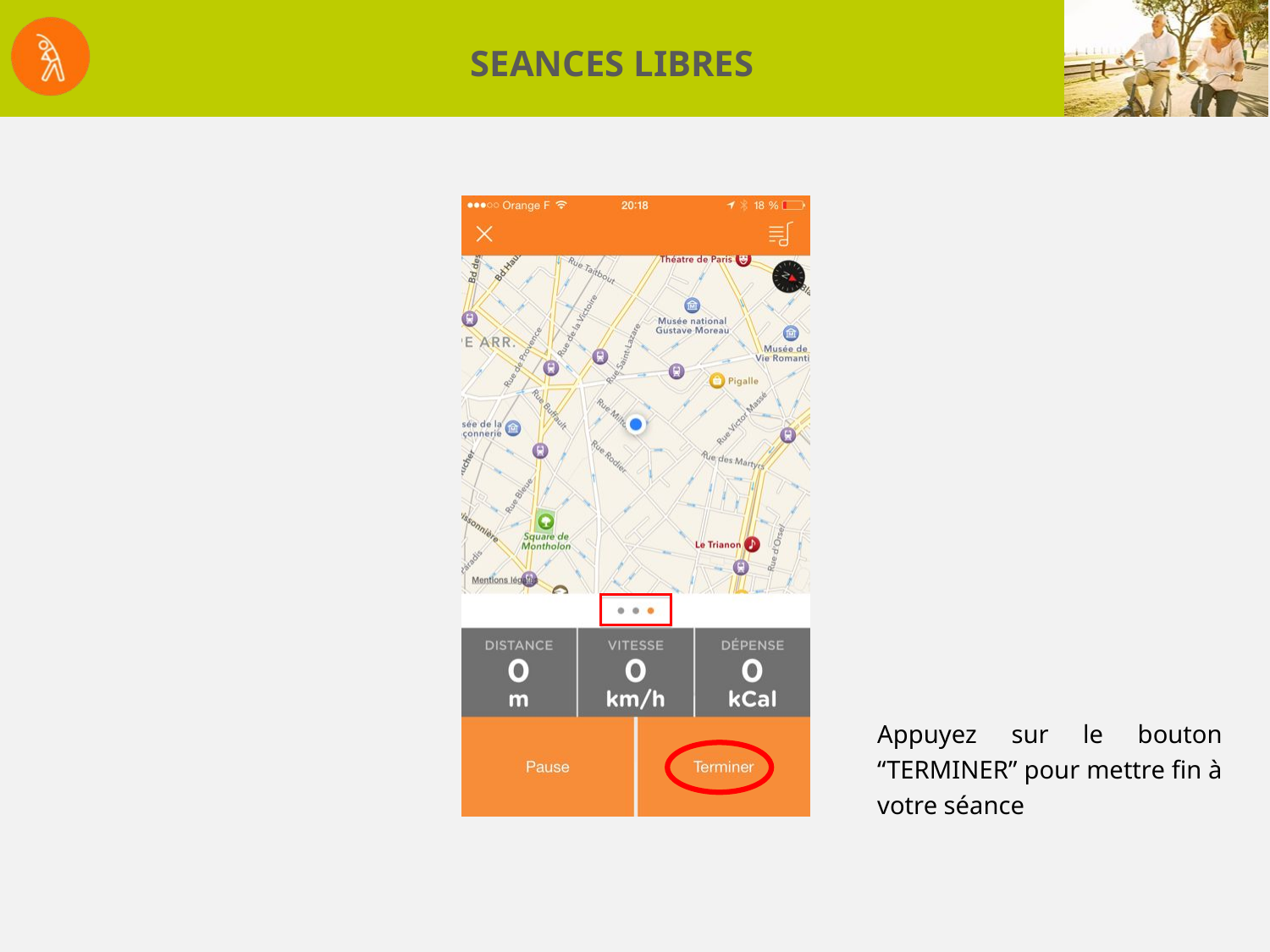

SEANCES LIBRES
Appuyez sur le bouton “TERMINER” pour mettre fin à votre séance

## Slide 15
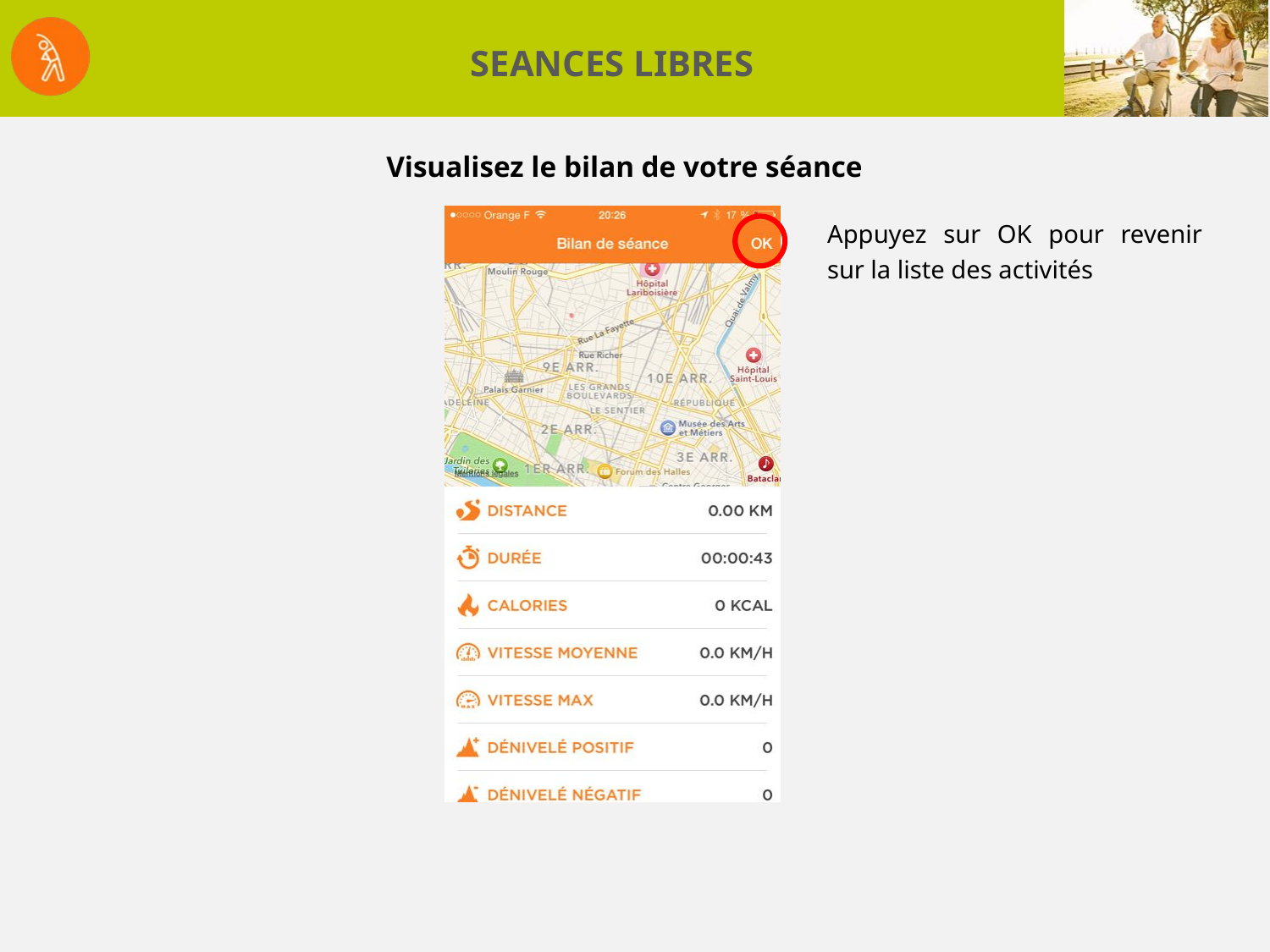

SEANCES LIBRES
Visualisez le bilan de votre séance
Appuyez sur OK pour revenir sur la liste des activités

## Slide 16
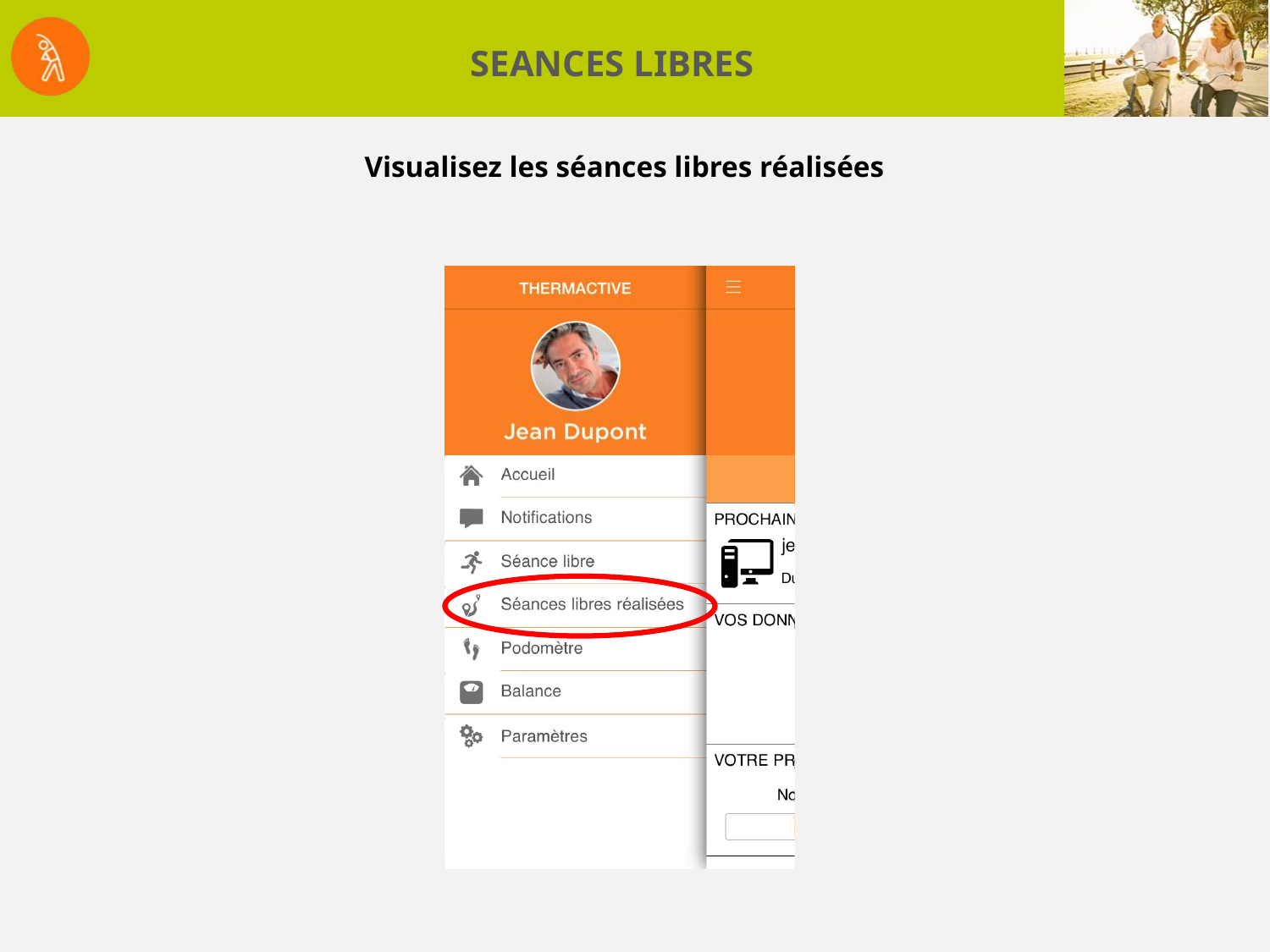

SEANCES LIBRES
Visualisez les séances libres réalisées

## Slide 17
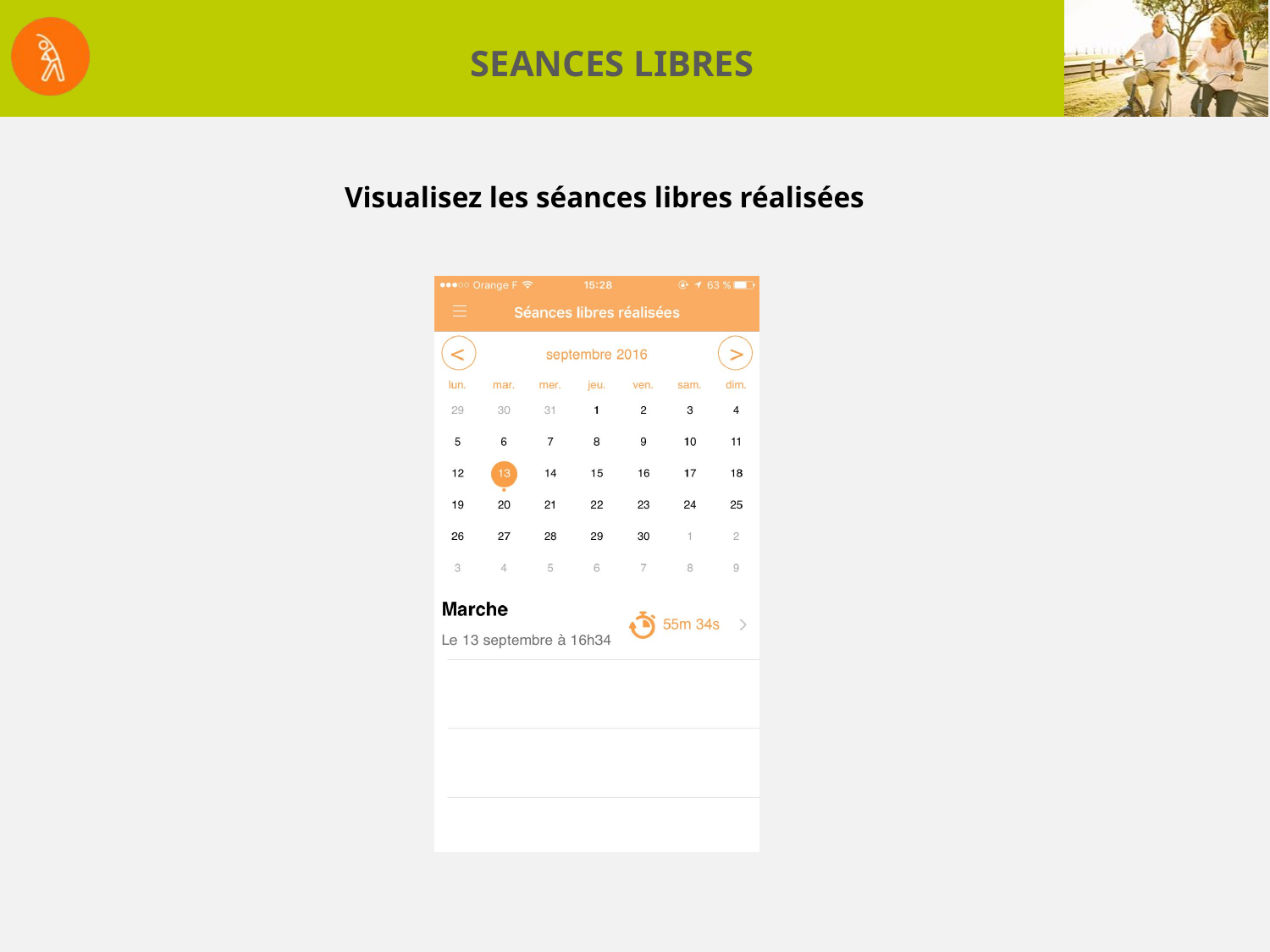

SEANCES LIBRES
Visualisez les séances libres réalisées

## Slide 18
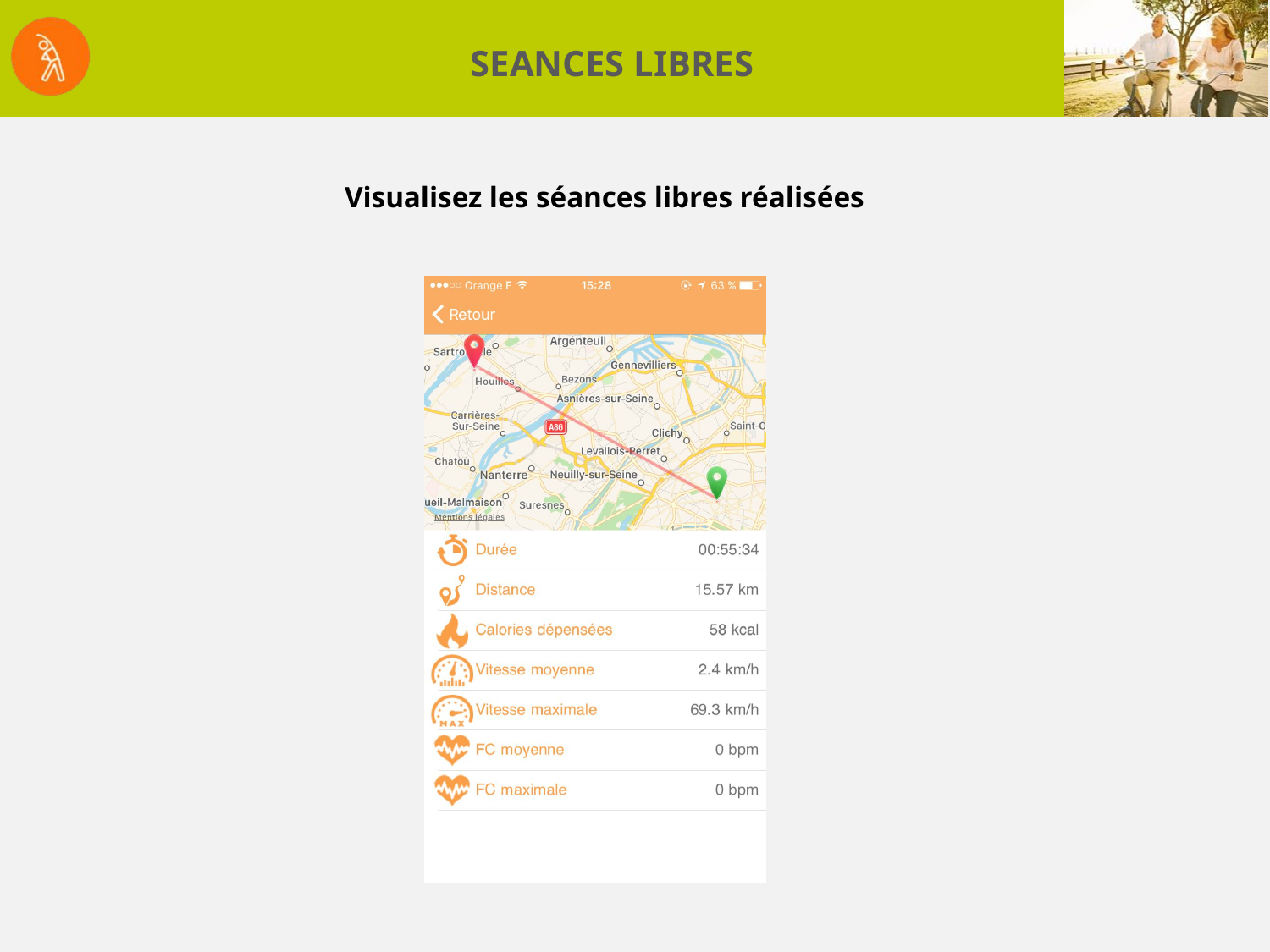

SEANCES LIBRES
Visualisez les séances libres réalisées

## Slide 19
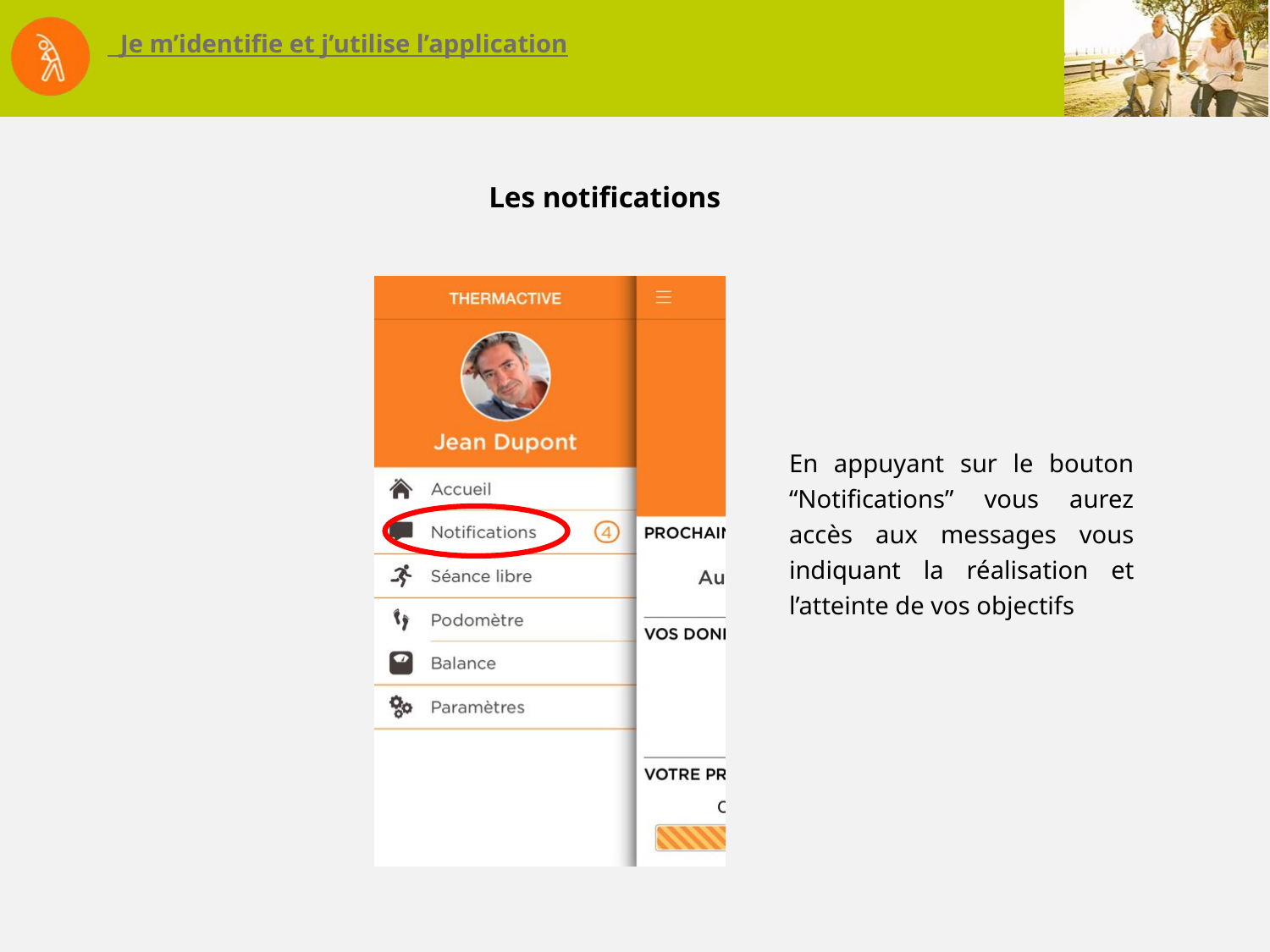

Je m’identifie et j’utilise l’application
Les notifications
En appuyant sur le bouton “Notifications” vous aurez accès aux messages vous indiquant la réalisation et l’atteinte de vos objectifs
